# From centripetal to centrifugal: pathological regression patterns after neoadjuvant or conversion therapy as markers of nodal risk and a framework for future research on individualized lymphadenectomy in gastric cancer

**DOI:** 10.3389/fimmu.2026.1766242

**Published:** 2026-04-13

**Authors:** Na Li, Penghui Liu, Jiwu Guo, Jizhen Wang, Gengyutong Zhao, Ziyuan Mou, Jie Mao

**Affiliations:** 1 Lanzhou University Second Clinical Medical College, Lanzhou, China; 2 Lanzhou University Second Hospital, Lanzhou, China; 3 Northeastern University, Boston, MA, United States

**Keywords:** gastric cancer, lymph node metastasis, lymphadenectomy, neoadjuvant therapy, tumor regression pattern

## Abstract

**Objective:**

To analyze the relationship between tumor regression patterns and ypN positivity and explore their implications for postoperative nodal-risk stratification after neoadjuvant or conversion therapy in advanced gastric cancer, while generating hypotheses for future individualized lymphadenectomy research.

**Methods:**

Tumor regression patterns were classified as centripetal, diffuse/mixed, or centrifugal. Clinical and pathological characteristics were compared using the Kruskal–Wallis and χ² tests. Using ypN positivity as the outcome, a multivariable logistic regression model was constructed. Sensitivity analyses were performed in the subgroup with ≥16 retrieved lymph nodes, after additional adjustment for ypT and Becker tumor regression grade (TRG), and in the non-pCR subgroup. Internal validation was performed using a 7:3 stratified random split and 10-fold cross-validation. Model performance was evaluated using the area under the receiver operating characteristic curve (AUC), 95% confidence intervals, calibration, and the Brier score. We additionally compared a baseline clinicopathological model with a combined model incorporating regression pattern to assess incremental predictive value.

**Results:**

Among 195 patients, 74 (38.0%) exhibited centripetal regression, 43 (22.1%) had diffuse/mixed regression, and 78 (40.0%) demonstrated centrifugal regression. Centripetal regression was characterized by low PRI, higher LRI and CER, and a very low ypN positivity rate (5.4%), whereas centrifugal regression showed the opposite pattern and the highest ypN positivity rate (75.6%); diffuse/mixed regression showed intermediate features (all p < 0.001). Multivariable analysis identified diffuse/mixed and centrifugal regression as the strongest independent predictors of ypN positivity. The apparent full-cohort model demonstrated an AUC of 0.875 (95% CI 0.826–0.922) with good calibration and a Brier score of 0.137. These associations remained robust after additional adjustment for ypT and Becker TRG and in the non-pCR subgroup. Internal validation showed acceptable performance, with a validation AUC of 0.826 in the 7:3 split-sample analysis and a pooled AUC of 0.822 in 10-fold cross-validation. Addition of regression pattern to the baseline clinicopathological model improved discrimination and reduced prediction error.

**Conclusion:**

Pathological regression patterns provide effective stratification of residual lymph node metastasis after neoadjuvant or conversion therapy. Centripetal regression indicates a very low residual nodal-risk phenotype, whereas centrifugal regression is associated with a heavier nodal burden. At present, regression patterns may be most appropriately used for postoperative risk assessment and multidisciplinary stratification. Their potential role in individualized lymphadenectomy should be viewed as a future translational direction requiring validated preoperative or intraoperative surrogate markers and prospective confirmation.

## 1 Introduction

Gastric cancer remains one of the leading causes of tumor-related morbidity and mortality worldwide (1). Despite significant advances in neoadjuvant therapy (NAT), prognostic stratification and surgical decision-making for gastric cancer remain challenges (2, 3). Current risk assessments and surgical strategies still rely mainly on clinical and pathological staging and the extent of lymph node dissection (4, 5), whereas accumulating evidence indicates that the degree of tumor regression and lymph node response are closely linked to long-term patient outcomes (6, 7).

Tumor regression following NAT not only reflects treatment efficacy but may also provide additional information for postoperative risk stratification and future translational research on surgical decision-making (8). Previous studies have focused on primary tumor regression grade (TRG) and its combined stratification with ypN status (6–8). However, systematic descriptions and analyses of regression “spatial patterns” remain limited. In clinical practice, various regression patterns can be observed, residual tumor may be predominantly central, predominantly peripheral, or scattered in a patchy distribution. The biological and clinical significance of these patterns remain unclear. Building on this observation, the study introduces a tripartite classification system consisting of centripetal, centrifugal, and diffuse/mixed regression patterns.

The extent and degree of lymph node involvement significantly influence patient survival and postoperative adjuvant treatment strategies in gastric cancer (9). Based on extensive clinical evidence, current guidelines generally recommend D2 lymphadenectomy as the standard scope for gastric cancer resection, emphasizing the importance of lymph node staging and the number of lymph nodes retrieved for prognosis (9, 10). However, the biological heterogeneity of post-treatment residual disease suggests that postoperative nodal risk may not be uniform across all patients after neoadjuvant therapy. In this context, a marker capable of capturing treatment-related spatial regression phenotypes may help refine postoperative risk stratification. At present, such information is more appropriately interpreted as a postoperative pathological reference for residual nodal-risk assessment, prognostic evaluation, and multidisciplinary treatment planning. In the longer term, if reliable preoperative or intraoperative surrogates of these regression phenotypes can be developed, this framework may also inform future research on individualized lymphadenectomy.

Research on tumor regression patterns has primarily focused on tumor regression grading (TRG) itself, or on combining TRG with ypN status and lymph node regression extent for prognostic stratification (6, 7, 10). Studies directly examining the relationship between spatial regression patterns and ypN status remain limited, with particularly scarce evidence systematically assessing how different regression patterns relate to residual lymph node metastasis. Therefore, this study aimed to explore whether different tumor regression patterns could independently predict postoperative lymph node positivity and provide a more refined framework for postoperative nodal-risk stratification after neoadjuvant or conversion therapy, while also generating hypotheses for future translational research on individualized lymphadenectomy.

## 2 Methods

### 2.1 Study design and patient selection

This study is a single-center, retrospective cohort study conducted at the Second Hospital of Lanzhou University. Patients who underwent gastrectomy following neoadjuvant or conversion therapy for gastric cancer between 2021 and 2025 were included.

#### 2.1.1 Inclusion criteria

(1) Histologically diagnosed as gastric adenocarcinoma; (2) Clinically staged as locally advanced gastric cancer and suitable for neoadjuvant or conversion therapy; (3) Underwent gastrectomy with curative intent after completion of neoadjuvant or conversion therapy; (4) Availability of complete staging and postoperative pathological data.

#### 2.1.2 Exclusion criteria

(1) Only received palliative or non-curative surgery; (2) Did not complete neoadjuvant or conversion therapy and curative surgery; (3) Missing clinical or pathological data.

A total of 195 patients were included in the final analysis cohort.

### 2.2 Neoadjuvant/conversion therapy

All patients received preoperative systemic therapy according to institutional practice and multidisciplinary team recommendations. Regimens were classified as chemotherapy alone (chemo), chemotherapy plus immunotherapy (chemo+IO), or chemotherapy plus immunotherapy and targeted therapy (chemo+IO+targeted). Chemotherapy was mainly platinum-based doublet or triplet regimens (e.g. SOX, FLOT), given for 2–7 cycles depending on regimen and response. Immunotherapy primarily consisted of immune checkpoint inhibitors, mainly programmed cell death protein 1 (PD-1) inhibitors, whereas targeted therapy was used in patients with actionable targets, such as human epidermal growth factor receptor 2 (HER2). The choice of regimen and treatment duration were determined by clinical staging, the patient’s general condition, and current treatment guidelines.

### 2.3 Surgical approach and lymph node dissection

After completing NAT, all patients underwent resectability assessment. Those deemed suitable for curative treatment received gastrectomy (total, distal, or proximal) according to tumor location and extent. All operations were performed by experienced gastric surgeons, and standard D2 lymphadenectomy was uniformly applied in accordance with the Japanese gastric cancer treatment guidelines. Postoperatively, the total and positive lymph node counts were recorded. For evaluating staging quality and conducting sensitivity analyses, patients were stratified by total lymph nodes into <16 and ≥16, a categorization used solely for analytic purposes and not reflecting differences in the extent of dissection. Resection margins were classified pathologically as R0 (microscopically negative) or R1 (microscopically positive).

### 2.4 Clinical and pathological data and staging

Baseline clinical and pathological variables included age, sex, body mass index (BMI), Eastern Cooperative Oncology Group (ECOG) performance status, primary tumor location (cardia, body, antrum), Lauren classification (intestinal, diffuse, mixed), histologic grade (moderate–low, moderate, low), and Borrmann type (II–IV). Pretreatment clinical stage (cT, cN, cM) was assessed according to the AJCC 8th edition criteria. Tumor biomarkers included HER2 status, microsatellite instability (MSI-H vs MSS), and programmed death-ligand 1 (PD-L1) expression, reported as combined positive score (CPS) and grouped as CPS <5 vs ≥5.

### 2.5 Pathological evaluation

All resected specimens were processed according to standard protocols, with tumor size, location, macroscopic type, histologic type and grade, margin status, and the number of total and positive lymph nodes recorded. Postoperative pathological stage (ypT, ypN) was assessed according to the AJCC 8th edition criteria. Tumor response was evaluated using the Becker TRG system (1a, 1b, 2, 3). Pathological complete response (pCR) was defined as ypT0N0 (Becker TRG 1a), and major pathological response (MPR) as <10% viable tumor cells in the primary tumor (Becker TRG 1a–1b).

### 2.6 Tumor regression patterns and quantitative regression indices

Tumor regression was assessed using postoperative resected specimens. Two experienced pathologists independently reviewed the slides without knowledge of the clinical information or prognosis and selected representative sections of the original tumor bed for analysis.

Based on the spatial distribution of residual tumor cells and fibrosis within the tumor bed, regression patterns were classified as centripetal, centrifugal, or diffuse/mixed. To objectively quantify these qualitative patterns, three semi-quantitative regression indices were introduced: Primary Residual Index (PRI), Lymph-node Regression Index (LRI), and Central–Edge Ratio (CER). Low PRI with LRI and CER close to 1.0 were centripetal; higher PRI with lower LRI and CER were centrifugal; and intermediate cases or discrepancies between quantitative indices and morphological features were diffuse/mixed. The detailed definitions of the three regression patterns and three indices can be found in the **Supplementary Materials**. Inter-observer agreement was evaluated using Cohen’s kappa, and discrepancies were resolved by joint review. Because regression patterns were determined from postoperative resected specimens, they were evaluated in this study as postoperative pathological markers rather than as tools for real-time modification of lymphadenectomy extent during the index operation.

### 2.7 Outcome measures

The primary outcome of this study was postoperative pathological lymph node positivity (ypN+), defined as the presence of at least one metastatic lymph node in the specimen (ypN≥1).

Secondary outcomes included ypN stage distribution (ypN0, ypN1, ypN2, ypN3), total number of positive lymph nodes, ypT stage, Becker TRG, pCR and MPR, and imaging or pathological downstaging, defined as a reduction in postoperative AJCC stage.

### 2.8 Statistical analysis

All statistical analyses were performed using SPSS (version 26.0, IBM Corp., Armonk, NY, USA) and R (version 4.3.2). Continuous variables were summarized as medians with interquartile ranges (IQRs) and compared across the three regression patterns using the Kruskal–Wallis test. Categorical variables were expressed as counts and percentages and compared using the χ² test. Missing data were handled using complete-case analysis because the proportion of missing values was low.

To assess whether regression pattern was independently associated with ypN positivity, multivariable logistic regression models were constructed with ypN status (positive vs negative) as the dependent variable. Covariates in the primary model were selected *a priori* based on clinical relevance, prior literature, and biological plausibility rather than univariable screening or stepwise procedures. Specifically, regression pattern was the main exposure of interest; cT and cN were included to reflect baseline tumor burden; Lauren classification and tumor location to capture biological heterogeneity; treatment regimen and PD-L1 status because of their potential association with treatment response; and total retrieved lymph node count to account for nodal staging adequacy. Univariable screening was not used for covariate selection because clinically important confounders may not meet arbitrary significance thresholds in univariable analyses. Results were reported as odds ratios (ORs) with 95% confidence intervals (CIs) and p-values. Pairwise *post-hoc* contrasts (centrifugal vs diffuse/mixed; Lauren mixed vs diffuse) were additionally examined in the full cohort and in the subgroup with ≥16 retrieved lymph nodes.

Multicollinearity was assessed using variance inflation factors (VIFs) and inspection of the correlation matrix. To assess the robustness of the multivariable model in the presence of potential multicollinearity, additional sensitivity analyses were performed using alternative model specifications, including a model excluding cT and a model with simplified binary coding of cT (cT2–3 vs cT4) and cN (cN0–1 vs cN2–3).

Additional sensitivity analyses were performed to evaluate whether regression pattern retained incremental value beyond the depth of primary tumor response. Specifically, the main multivariable logistic regression model was refitted after additional adjustment for ypT and, separately, for Becker TRG. Because pCR was defined as ypT0N0 and therefore incorporates nodal status by definition, pCR was not included as a covariate in the model for ypN positivity. Instead, the main multivariable analysis was repeated in the non-pCR subgroup. In addition, likelihood-ratio tests were used to compare nested models with and without regression pattern after adjustment for ypT or Becker TRG, in order to assess the incremental contribution of regression pattern to the prediction of ypN positivity.

To directly assess the incremental predictive value of regression pattern beyond conventional clinicopathological predictors available in the dataset, we constructed a baseline model including cT, cN, Lauren classification, tumor location, treatment regimen, PD-L1 status, and total retrieved lymph node count, and then compared it with a combined model additionally including regression pattern. Improvement in model performance was assessed using likelihood-ratio testing, changes in AUC and Brier score, and the continuous net reclassification improvement (NRI) and integrated discrimination improvement (IDI). These analyses were further examined in internal validation procedures.

Model performance was evaluated by the area under the receiver operating characteristic curve (AUC) and by calibration, using observed–predicted plots by deciles of risk and the Hosmer–Lemeshow goodness-of-fit test. All multivariable analyses were repeated in patients with ≥16 lymph nodes as a sensitivity analysis. Marginal predicted probabilities of ypN positivity were derived from the primary model for different regression patterns (and Lauren types) while holding other covariates constant, and displayed as point estimates with 95% CIs. All tests were two-sided, and p<0.05 was considered statistically significant. Given the exploratory nature of the study, no formal adjustment for multiple comparisons was applied.

To further assess model stability and reduce the risk of optimistic performance estimation, internal validation was additionally performed using a 7:3 stratified random split and 10-fold stratified cross-validation. Model discrimination was assessed using the area under the receiver operating characteristic curve (AUC) with 95% confidence intervals, and calibration was evaluated using the Hosmer–Lemeshow test, calibration plots, and the Brier score. In the split-sample analysis, the model was developed in the training set and evaluated in the validation set. In 10-fold cross-validation, pooled out-of-fold predictions were used to estimate cross-validated discrimination and calibration performance. To further explore the shape of the associations between the semi-quantitative regression indices (PRI, LRI, and CER) and ypN positivity, exploratory restricted cubic spline (RCS) analyses were performed, and the corresponding curves are presented in the **Supplementary Materials**. To address potential confounding by treatment timing, the treatment-to-surgery interval was additionally examined in sensitivity analyses both as a continuous variable and as a prespecified categorical variable (<12, 12–16, and >16 weeks). Because lymph node ratio (LNR) is derived from postoperative nodal counts and therefore lies downstream of ypN status, it was not included as a confounder in the primary model; instead, LNR was calculated postoperatively and compared descriptively across regression patterns overall and within the ypN-positive subgroup.

## 3 Results

### 3.1 Patient characteristics

A total of 195 patients were included in the analysis. Among these, 162 patients had ≥16 lymph nodes and were included in subsequent sensitivity analyses (**Supplementary Figure 1**). Baseline characteristics are shown in **Supplementary Table 1**. The overall median age was 59 years, with 169 patients (86.7%) being male. The median BMI was 23.1 kg/m², and 92.3% of patients had an ECOG score of 0–1. Regarding therapy regimen, 47 patients (24.1%) received chemo, 100 patients (51.3%) received chemo+IO, and 48 patients (24.6%) received chemo+IO+targeted. The median number of treatment cycles was 4, with the median time interval from the start treatment to surgery being 14 weeks.

In terms of tumor anatomical location, 58 patients (29.7%) in the gastric body, 67 patients (34.4%) in the antrum, and 70 patients (35.9%) in the cardia. According to Lauren classification, 96 patients (49.2%) were intestinal-type, 71 patients (36.4%) were diffuse-type, and 28 patients (14.4%) were mixed-type. Regarding Histologic grade, 111 patients (56.9%) had Moderate-Low Grading, while 79 patients (40.5%) had Low Grading. In Borrmann type, 165 patients (84.6%) had type III, 18 patients (9.2%) had type IV, and 12 patients (6.2%) had type II.

Pre-treatment clinical staging showed 7 patients (3.6%) with cT2, 94 patients (48.2%) with cT3, and 94 patients (48.2%) with cT4. Regarding clinical N stage, 15 patients (7.7%) were cN0, 45 patients (23.1%) were cN1, 89 patients (45.6%) were cN2, and 46 patients (23.6%) were cN3. Most patients were cM0 (178 patients, 91.3%), though 17 patients (8.7%) had limited cM1 metastases, who were included in the conversion therapy strategy following multidisciplinary team discussion. HER2 scores showed 148 patients (75.9%) with a score of 0, 22 patients (11.3%) with 1+, 10 patients (5.1%) with 2+, 13 patients (6.7%) with 3+, and 2 patients (1.0%) with 4 +. MSI-H was observed in only 7 patients (3.6%), while the remaining 188 patients (96.4%) were MSS. In terms of PD-L1 expression, 82 patients (42.0%) had CPS ≥5, and 113 patients (58.0%) had CPS <5. Additionally, 13 patients (6.7%) had special tumor components.

All 195 patients underwent gastrectomy with D2 lymphadenectomy, with 102 patients (52.3%) undergoing total gastrectomy, 67 patients (34.4%) undergoing distal gastrectomy, and 26 patients (13.3%) undergoing proximal gastrectomy. The R0 resection rate was 99.5% (194 patients), with only 1 patient (0.5%) having R1 resection. The median perioperative hospital stay was 14 days (**Supplementary Table 1**).

### 3.2 Tumor regression patterns, quantitative regression indices, and reproducibility

Tumor regression patterns were classified as centripetal in 74 patients (38.0%), diffuse/mixed in 43 patients (22.1%), and centrifugal in 78 patients (40.0%). Overall, the median PRI was 0.35, the median LRI was 1.00, and the median CER was 0.68. The consistency of regression pattern classification was high, with an overall median kappa of 0.90. The highest consistency was observed for the centripetal pattern (**Table 1**).

**Table 1:** Comparison of continuous variables according to tumor regression pattern.

Variable	Centripetal (n=74)	Diffuse/mixed (n=43)	Centrifugal (n=78)	H	P
Age	59.500(55.8,67.0)	57.000(51.0,64.0)	60.000(53.0,67.3)	4.272	0.118
BMI	23.200(22.2,24.6)	23.000(22.4,24.2)	23.050(22.1,23.9)	1.444	0.486
preoperative cycles	4.000(3.0,4.0)	4.000(3.0,4.0)	4.000(3.0,4.0)	2.410	0.300
Interval from NAT start to surgery (weeks)	14.000(12.0,16.0)	15.000(10.0,16.0)	15.000(10.0,16.0)	0.001	1.000
Length of hospital stay (days)	14.000(11.0,17.0)	14.000(12.0,18.0)	14.000(12.0,16.0)	1.695	0.429
Total LN Count	22.000(18.0,28.3)	22.000(17.0,24.0)	24.000(17.0,28.0)	0.501	0.778
Positive LN Count	0.000(0.0,0.0)	0.000(0.0,2.0)	3.000(0.8,7.3)	79.026	0.000**
PRI	0.000(0.0,0.1)	0.350(0.3,0.4)	0.700(0.6,0.9)	160.065	0.000**
LRI	1.000(1.0,1.0)	0.950(0.7,1.0)	0.620(0.2,0.9)	68.541	0.000**
CER	1.000(1.0,1.0)	0.670(0.6,0.8)	0.450(0.3,0.6)	157.247	0.000**
kappa	1.000(1.0,1.0)	0.870(0.8,0.9)	0.825(0.8,0.9)	105.350	0.000**

Data are presented as median (interquartile range, IQR). H statistic is derived from the Kruskal–Wallis test. Abbreviations: NAT, neoadjuvant/conversion therapy; LN, lymph node; PRI, primary residual index; LRI, lymph-node regression index; CER, central–edge ratio.** p<0.01.

Significant differences in PRI, LRI, and CER were observed across the three groups (all p < 0.001). For centripetal tumors, PRI was close to 0, and both LRI and CER were close to 1, indicating a very low residual tumor burden with the residual tumor primarily concentrated in the center of the tumor bed. In contrast, centrifugal tumors exhibited significantly higher PRI, lower LRI and CER, suggesting a larger residual tumor burden with peripheral predominance. Diffuse/mixed tumors displayed intermediate values for these indices. These results support the biological differences between the three regression patterns and demonstrate good reproducibility in the classification of regression patterns.

No significant differences were found between the regression patterns with regard to age, BMI, number of preoperative treatment cycles, time interval from treatment to surgery, length of hospital stay, or total number of lymph nodes (all p > 0.05).

Exploratory restricted cubic spline analyses were performed to assess potential nonlinear associations between PRI, LRI, CER, and ypN positivity (**Supplementary Figure 2**). PRI and LRI showed significant nonlinear associations with ypN positivity, whereas CER showed a significant overall association with a weaker nonlinear component. These findings further suggest that the semi-quantitative regression indices capture structured pathological gradients related to residual nodal risk.

### 3.3 Association between regression patterns and clinical/pathological features

Tumor location was distributed differently among the regression patterns (p = 0.014): centripetal regression was more common in distal (antrum) tumors, whereas diffuse/mixed and centrifugal regression were more frequently observed in the cardia tumors. Lauren classification also showed significant differences (p < 0.001). Among centripetal tumors, 63/74 patients (85.1%) had intestinal-type, and only 8/74 patients (10.8%) had diffuse-type. In contrast, diffuse/mixed and centrifugal tumors were predominantly diffuse-type (20/43 [46.5%] and 43/78 [55.1%]), with lower proportions of intestinal-type tumors (14/43 [32.6%] and 19/78 [24.4%]). Borrmann classification showed a similar pattern (p < 0.001), with a higher proportion of type II tumors in the centripetal group (about 14.9%), and a higher proportion of type IV tumors in the centrifugal group (about 14.1%) (**Table 2**).

**Table 2:** Comparison of clinicopathologic characteristics and treatment regimens by tumor regression pattern.

Variable	Category	Centripetal (n=74)	Diffuse/mixed (n=43)	Centrifugal (n=78)	χ2	P
Sex	Female	10(13.51)	9(20.93)	7(8.97)	3.432	0.180
	Male	64(86.49)	34(79.07)	71(91.03)		
ECOG status	0	7(9.46)	4(9.30)	6(7.69)	1.019	0.907
	1	63(85.14)	35(81.40)	65(83.33)		
	2	4(5.41)	4(9.30)	7(8.97)		
Tumor location	body	21(28.38)	8(18.60)	29(37.18)	12.485	0.014*
	antrum	32(43.24)	11(25.58)	24(30.77)		
	cardia	21(28.38)	24(55.81)	25(32.05)		
Lauren classification	diffuse	8(10.81)	20(46.51)	43(55.13)	62.459	0.000**
	mixed	3(4.05)	9(20.93)	16(20.51)		
	intestinal	63(85.14)	14(32.56)	19(24.36)		
Histologic grade	Moderate-Low Grade	47(63.51)	23(53.49)	41(52.56)	7.466	0.113
	Moderate Grade	4(5.41)	0(0.00)	1(1.28)		
	Low Grade	23(31.08)	20(46.51)	36(46.15)		
Borrmann type	II	11(14.86)	0(0.00)	1(1.28)	22.817	0.000**
	III	62(83.78)	37(86.05)	66(84.62)		
	IV	1(1.35)	6(13.95)	11(14.10)		
cT	2	4(5.41)	0(0.00)	3(3.85)	5.972	0.201
	3	41(55.41)	21(48.84)	32(41.03)		
	4	29(39.19)	22(51.16)	43(55.13)		
cN	0	7(9.46)	4(9.30)	4(5.13)	4.206	0.649
	1	15(20.27)	13(30.23)	17(21.79)		
	2	34(45.95)	15(34.88)	40(51.28)		
	3	18(24.32)	11(25.58)	17(21.79)		
cM	0	67(90.54)	40(93.02)	71(91.03)	0.221	0.895
	1	7(9.46)	3(6.98)	7(8.97)		
MSI status	MSI-H	4(5.41)	1(2.33)	2(2.56)	1.141	0.565
	MSS	70(94.59)	42(97.67)	76(97.44)		
PD-L1 status	CPS<5	32(43.24)	28(65.12)	53(67.95)	10.674	0.005**
	CPS≥5	42(56.76)	15(34.88)	25(32.05)		
Type of gastrectomy	total	35(47.30)	23(53.49)	44(56.41)	3.918	0.417
	proximal	8(10.81)	8(18.60)	10(12.82)		
	distal	31(41.89)	12(27.91)	24(30.77)		
Resection margin status	R0	74(100.00)	43(100.00)	77(98.72)	1.508	0.471
	R1	0(0.00)	0(0.00)	1(1.28)		
Treatment regimen	chemo	14(18.92)	13(30.23)	20(25.64)	7.708	0.103
	chemo+IO	34(45.95)	23(53.49)	43(55.13)		
	chemo+IO+targeted	26(35.14)	7(16.28)	15(19.23)		

Data are presented as n (%). χ² statistics are derived from the chi-square test. *p < 0.05, **p < 0.01. Abbreviations: ECOG, Eastern Cooperative Oncology Group; MSI, microsatellite instability; PD-L1, programmed death-ligand 1; CPS, combined positive score; NAT, neoadjuvant/conversion therapy.

PD-L1 status also differed across regression patterns (p = 0.005). In centripetal tumors, 42/74 patients (56.8%) had CPS ≥5, while diffuse/mixed and centrifugal tumors were predominantly CPS <5 (65.1% and 68.0%, respectively). Although no statistical significance was reached in terms of therapy regimens (p = 0.103), a trend was observed: centripetal tumors were more likely to receive chemo+IO+targeted therapy, while diffuse/mixed and centrifugal tumors were more commonly treated with chemo or chemo+IO.

No significant differences in Sex, ECOG status, histologic grade, clinical T/N stage, cM status, MSI status, type of gastrectomy, or R status among the three groups.(all p > 0.05).

### 3.4 Regression patterns and pathological response of primary tumors

There were significant differences in the distribution of ypT among the groups (p < 0.001). In the centripetal group, 40/74 patients (54.1%) had ypT0, and 20/74 patients (27.0%) had ypT1, resulting in a total of 60/74 patients (81.1%) achieving ypT0–1. In contrast, only 1/43 patients (2.3%) in the diffuse/mixed group had ypT0, with the majority being ypT2–3 (32/43, 74.4%). In the centrifugal group, ypT3–4 was predominant (64/78, 82.1%).

Becker TRG revealed a more distinct gradient change (p < 0.001). Among centripetal tumors, 71/74 patients (96.0%) were grade 1, 3/74 patients (4.0%) were grade 2. In the diffuse/mixed group, only 7/43 patients (16.3%) were grade 1, 33/43 patients (76.7%) were grade 2, and 3/43 patients (7.0%) were grade 3. The centrifugal group had no grade 1 cases, with 16/78 patients (20.5%) were grade 2, and 62/78 patients (79.5%) were grade 3.

When stratified by MPR, 71/74 patients (95.9%) in the centripetal group met the MPR, with 39/74 patients (52.7%) achieving pCR. In the diffuse/mixed group, 7/43 patients (16.3%) achieved MPR, and only 1/43 patient (2.3%) achieved pCR. In the centrifugal group, there were no cases of MPR or pCR (all 78 patients had >10% residual tumor burden; p < 0.001).

There were also significant differences in downstaging (p < 0.001). All centripetal cases (74/74, 100%) achieved downstaging, while the downstaging rate in the diffuse/mixed group was 40/43 patients (93.0%). In the centrifugal group, only 43/78 patients (55.1%) achieved downstaging, with 25/78 patients (32.1%) showing disease progression after treatment (**Table 3**).

**Table 3:** Pathological tumor response and downstaging according to regression pattern.

Variable	Category	Centripetal (n=74)	Diffuse/mixed (n=43)	Centrifugal (n=78)	χ2	P
ypT	0	40(54.05)	1(2.33)	0(0.00)	129.107	0.000**
	1	20(27.03)	6(13.95)	4(5.13)		
	2	8(10.81)	10(23.26)	10(12.82)		
	3	5(6.76)	22(51.16)	37(47.44)		
	4	1(1.35)	4(9.30)	27(34.62)		
ypN	0	70(94.59)	23(53.49)	19(24.36)	100.656	0.000**
	1	3(4.05)	17(39.53)	19(24.36)		
	2	0(0.00)	3(6.98)	16(20.51)		
	3	1(1.35)	0(0.00)	24(30.77)		
TRG_Becker	1	71(95.95)	7(16.28)	0(0.00)	234.362	0.000**
	2	3(4.05)	33(76.74)	16(20.51)		
	3	0(0.00)	3(6.98)	62(79.49)		
Pathological Response	MPR	32(43.24)	6(13.95)	0(0.00)	161.056	0.000**
	pCR	39(52.70)	1(2.33)	0(0.00)		
	No	3(4.05)	36(83.72)	78(100.00)		
Downgrading	No	0(0.00)	2(4.65)	10(12.82)	55.184	0.000**
	progression	0(0.00)	1(2.33)	25(32.05)		
	Yes	74(100.00)	40(93.02)	43(55.13)		

Data are presented as n (%). χ² statistics are derived from the chi-square test. **p < 0.01. Definitions: ypT/ypN, post-treatment pathologic T/N stage; TRG_Becker, Becker tumor regression grade (1–3); MPR (major pathological response), Becker grade 1a–1b; pCR, pathological complete response (ypT0N0); Downgrading, post-treatment AJCC stage lower than baseline clinical stage.

### 3.5 Regression patterns and lymph node burden

Regression patterns showed a significant stepwise gradient in lymph node status (**Supplementary Figure 3**). In the entire cohort, 112 patients (57.5%) were ypN0, 39 patients (20.0%) were ypN1, 19 patients (9.7%) were ypN2, and 25 patients (12.8%) were ypN3. In the centripetal regression group, 70/74 patients (94.6%) were ypN0, and only 4 patients (5.4%) had any lymph node metastasis. In contrast, 20/43 patients (46.5%) in the diffuse/mixed group and 59/78 patients (75.6%) in the centrifugal group were ypN≥1. ypN staging exhibited a monotonic increase from centripetal to diffuse/mixed to centrifugal regression patterns (**Table 3**).

The number of positive lymph nodes showed significant differences across the regression patterns (p < 0.001). The median number of positive lymph nodes in the centripetal group was 0, while in the diffuse/mixed group, the median was 0, but the distribution was slightly skewed to the right. In the centrifugal group, the median was 3, indicating that the burden of positive lymph nodes increased progressively from centripetal to centrifugal regression patterns. The total number of lymph nodes retrieved was similar across the three groups (median 22–24 nodes, p = 0.778), suggesting that the gradient difference in lymph node burden was not due to an imbalance in the extent of lymph node dissection or the number of lymph nodes retrieved (**Table 1**).

### 3.6 Multivariate analysis of regression patterns and ypN positivity

For transparency, univariable logistic regression analyses were additionally performed for candidate predictors of ypN positivity, and the results are provided in **Supplementary Table 2**. The multivariate logistic regression model was constructed with ypN positivity (ypN≥1) as the dependent variable. Covariates included regression pattern, Lauren classification, treatment regimen, tumor location, clinical T and N stages, PD-L1 status, and total number of lymph nodes.

**Figure 1:**
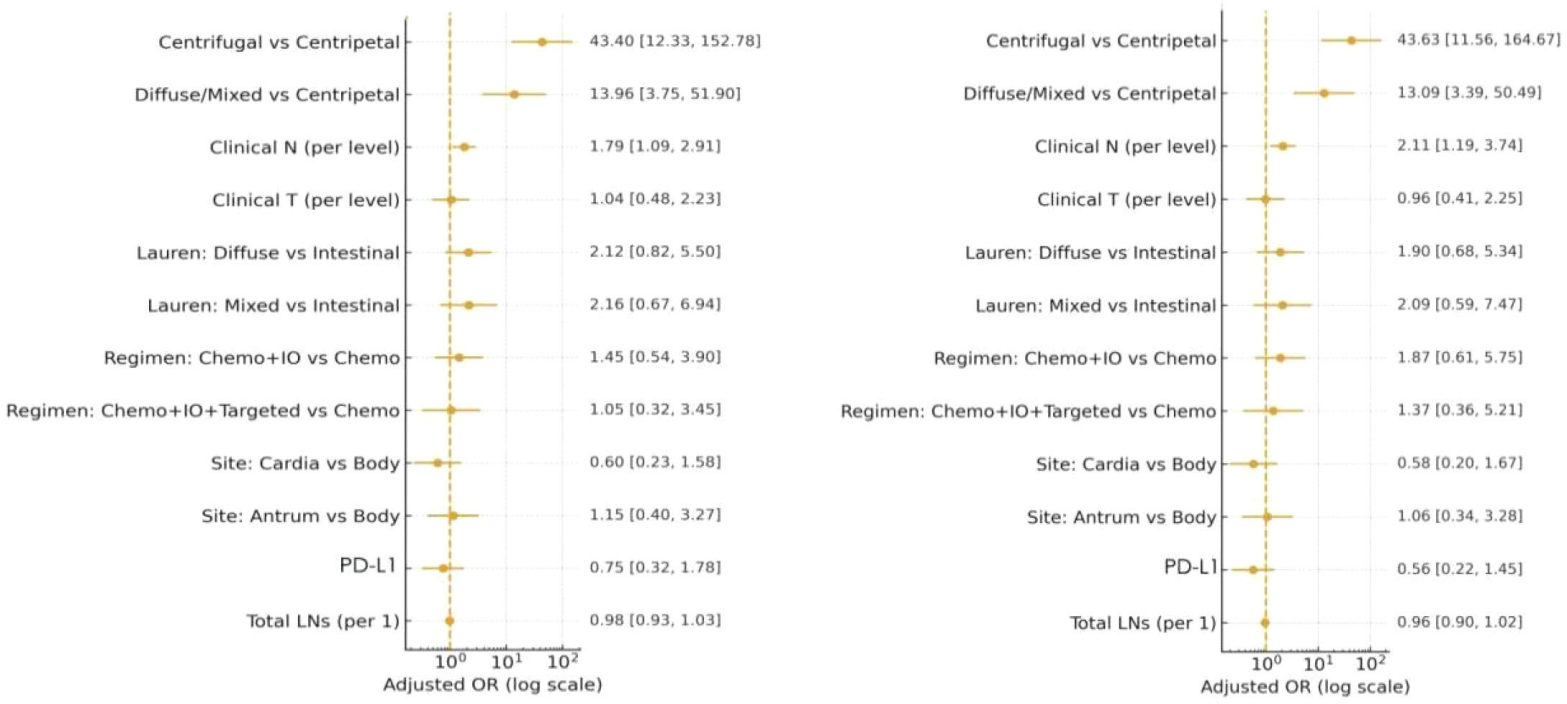
Adjusted odds ratios for ypN positivity from the main multivariable logistic regression model in patients with full cohort. Adjusted odds ratios for ypN positivity from the multivariable logistic regression model in patients with ≥16 lymph nodes.

In the full cohort analysis (**Figure 1**), regression pattern was the most significant determinant of ypN positivity. With centripetal regression as the reference, the adjusted OR for diffuse/mixed regression was 13.96 (95% CI 3.76–51.90, p < 0.001), and the OR for centrifugal regression was 43.40 (95% CI 12.33–152.78, p < 0.001). Clinical N stage was the other covariate independently associated with ypN positivity: for each increase in one cN stage, the risk of ypN positivity increased by approximately 1.79 times (OR 1.79, 95% CI 1.09–2.91, p = 0.020). No significant associations between ypN positivity and Lauren classification, treatment regimen, tumor location, PD-L1 status, or total lymph nodes (all p > 0.05) (**Table 4**).

**Table 4:** Multivariable logistic regression analysis of predictors of ypN positivity in the overall cohort.

Variable	OR	95% CI	P
Regression: Diffuse/Mixed vs Centripetal	13.96	3.755 ~ 51.904	0.000**
Regression: Centrifugal vs Centripetal	43.401	12.329 ~ 152.775	0.000**
Lauren: Diffuse vs Intestinal	2.122	0.819 ~ 5.497	0.121
Lauren: Mixed vs Intestinal	2.156	0.67 ~ 6.937	0.198
Regimen: chemo+IO vs chemo	1.446	0.536 ~ 3.898	0.466
Regimen: chemo+IO+targeted vs chemo	1.052	0.32 ~ 3.453	0.934
Location: Antrum vs Body	1.15	0.404 ~ 3.269	0.794
Location: Cardia vs Body	0.596	0.225 ~ 1.578	0.298
cT	1.04	0.484 ~ 2.235	0.92
cN	1.785	1.094 ~ 2.912	0.02*
PD-L1	0.752	0.317 ~ 1.782	0.517
Total LN Count	0.978	0.93 ~ 1.028	0.386

OR, odds ratio; CI, confidence interval; LN, lymph node; chemo, chemotherapy; IO, immunotherapy. OR > 1 indicates increased odds of ypN positivity compared with the reference category. *p < 0.05, **p < 0.01.

The sensitivity analysis was repeated in the subgroup of patients with ≥16 lymph nodes (**Figure 1**). The results showed that the effect of regression patterns remained largely unchanged: Compared to centripetal regression, the OR for diffuse/mixed regression was 13.09 (95% CI 3.39–50.49, p < 0.001), and the OR for centrifugal regression was 43.63 (95% CI 11.56–164.67, p < 0.001). Clinical N stage remained an independent risk factor for ypN positivity (OR 2.11, 95% CI 1.19–3.74, p = 0.012), while no significant associations were found for other covariates (**Supplementary Table 3**).

To assess the potential influence of multicollinearity related to baseline clinical staging, we performed additional analyses using alternative model specifications. After excluding cT from the primary multivariable model, regression pattern remained the strongest independent predictor of ypN positivity. Compared with centripetal regression, the adjusted OR was 13.97 (95% CI 3.76–51.87, p < 0.001) for diffuse/mixed regression and 43.45 (95% CI 12.36–152.78, p < 0.001) for centrifugal regression. Overall model performance remained stable (AUC 0.875; Hosmer–Lemeshow p = 0.304). We further simplified baseline clinical staging by recoding cT as cT2–3 vs cT4 and cN as cN0–1 vs cN2–3. Under this alternative specification, regression pattern remained robustly associated with ypN positivity, with adjusted ORs of 12.70 (95% CI 3.50–46.09, p < 0.001) for diffuse/mixed regression and 37.81 (95% CI 11.07–129.15, p < 0.001) for centrifugal regression. Model discrimination and calibration also remained acceptable (AUC 0.869; Hosmer–Lemeshow p = 0.105). These alternative model specifications are presented in **Supplementary Tables 4, 5**, respectively.

### 3.7 Paired comparisons and additional value beyond Lauren classification

*Post-hoc* regression contrasts were performed within the multivariate model to directly compare centrifugal and diffuse/mixed regression patterns. In the full cohort, the risk of ypN positivity for centrifugal regression was approximately three times that of diffuse/mixed regression (OR 3.11, 95% CI 1.33–7.27, p = 0.009). A similar result was in the subgroup with ≥16 lymph nodes (OR 3.33, 95% CI 1.30–8.53, p = 0.012) (**Supplementary Figure 4**).

In contrast, paired comparisons between Lauren mixed and diffuse types did not show significant differences in ypN positivity risk. In the full cohort, the OR for mixed-type compared to diffuse-type was 1.02 (95% CI 0.34–3.05, p = 0.98); in the subgroup with ≥16 lymph nodes, the OR was 1.10 (95% CI 0.34–3.57, p = 0.87) (**Supplementary Figure 5**).

### 3.8 Model performance, multicollinearity diagnostics, and marginal predicted probabilities

Multicollinearity diagnostics showed that the VIFs for categorical variables (regression pattern, Lauren classification, treatment regimen, tumor location, and PD-L1) were all within an acceptable range (<3.5), whereas the VIFs for cN and total retrieved lymph node count were moderately elevated (approximately 6.4 and 9.1, respectively), and the VIF for cT was high (approximately 15.8). Given this finding, we performed additional alternative model specifications to assess the robustness of the primary multivariable model. Importantly, after excluding cT and after simplifying cT/cN coding, the estimated effects of regression pattern and overall model performance remained materially unchanged, supporting that the main findings were not driven by the inclusion or coding of cT. Therefore, these clinically relevant variables were retained in the primary model, and the alternative model results are presented in **Supplementary Tables 4, 5**.

**Figure 2:**
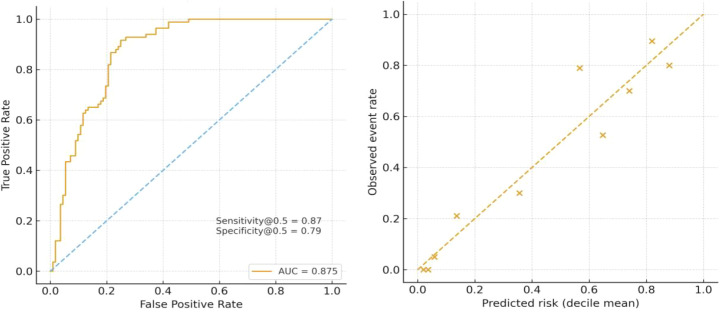
ROC curve of the main multivariable logistic model for ypN positivity in the full cohort. Calibration of the multivariable logistic regression model for ypN positivity in the full cohort (Hosmer–Lemeshow p = 0.304).

**Figure 3:**
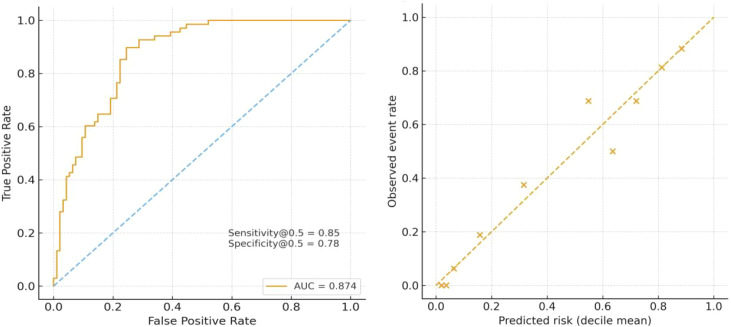
ROC curve of the multivariable logistic model for ypN positivity in patients with ≥16 lymph nodes. Calibration of the multivariable logistic regression model for ypN positivity in patients with ≥16 lymph nodes (Hosmer–Lemeshow p = 0.866).

The primary multivariate model demonstrated good discriminatory ability for ypN positivity, with an AUC of 0.875. At a prediction probability threshold of 0.5, the sensitivity was 86.7%, specificity was 78.6%, and overall accuracy was 82.1%. The Hosmer–Lemeshow test indicated good model calibration (χ² = 9.48, df = 8, p = 0.30), and the calibration curve showed a high degree of agreement between observed and predicted values across deciles of predicted risk (**Figure 2**). In the subgroup with ≥16 lymph nodes, the model performance remained nearly identical: the AUC was 0.874, sensitivity was 85.3%, specificity was 77.7%, and overall accuracy was 80.9%. The Hosmer–Lemeshow test indicated excellent calibration (χ² = 3.90, df = 8, p = 0.87), and the observed and predicted event rate curves were nearly overlapping (**Figure 3**).

The marginal predicted probabilities calculated based on the primary logistic model showed a clear stepwise gradient of ypN positivity risk across different regression patterns (**Supplementary Figure 6**). Keeping other covariate distributions constant, the adjusted predicted probability for ypN ≥1 was approximately 5%–10% for centripetal regression, 45%–50% for diffuse/mixed regression, and 70% for centrifugal regression. In contrast, the ypN positivity predicted probabilities for different Lauren types were roughly in the 35%–50% range, with substantial overlap in the 95% CI for all three groups (**Supplementary Figure 7**).

### 3.9 Additional sensitivity analyses beyond primary tumor response depth

To determine whether regression pattern provided information beyond the depth of primary tumor response, we performed additional sensitivity analyses. After further adjustment for ypT in the main multivariable model, regression pattern remained independently associated with ypN positivity. Compared with centripetal regression, the adjusted OR was 7.41 (95% CI 1.82–30.19, p = 0.005) for diffuse/mixed regression and 17.86 (95% CI 4.39–72.66, p < 0.001) for centrifugal regression. ypT was also independently associated with ypN positivity (OR 1.77, 95% CI 1.12–2.78, p = 0.014)(**Supplementary Table 6**).

Similarly, after further adjustment for Becker TRG, the association between regression pattern and ypN positivity remained significant, with adjusted ORs of 15.48 (95% CI 3.50–68.42, p < 0.001) for diffuse/mixed regression and 55.05 (95% CI 7.11–426.11, p < 0.001) for centrifugal regression, whereas Becker TRG itself was not independently associated with ypN positivity (OR 0.86, 95% CI 0.31–2.38, p = 0.771)(**Supplementary Table 7**).

Because pCR was defined as ypT0N0 and therefore could not be directly included as an adjustment variable, we repeated the main multivariable analysis in the non-pCR subgroup (n = 155). In this restricted cohort, regression pattern remained an independent predictor of ypN positivity, with adjusted ORs of 7.60 (95% CI 2.02–28.62, p = 0.003) for diffuse/mixed regression and 22.99 (95% CI 6.46–81.84, p < 0.001) for centrifugal regression. Likelihood-ratio testing further confirmed that regression pattern added significant incremental information beyond ypT (p = 5.19 × 10^-5^) and beyond Becker TRG (p = 9.13 × 10^-5^)(**Supplementary Table 8**).

### 3.10 Incremental predictive value beyond conventional predictors

To directly evaluate the incremental predictive value of regression pattern beyond conventional clinicopathological variables, we compared a baseline model including cT, cN, Lauren classification, tumor location, treatment regimen, PD-L1 status, and total retrieved lymph node count with a combined model additionally incorporating regression pattern. In the full cohort, addition of regression pattern improved model discrimination from an AUC of 0.777 to 0.875 and reduced the Brier score from 0.188 to 0.137. Nested likelihood-ratio testing confirmed a significant improvement in model fit (p = 2.45 × 10^-^¹²).

This incremental value remained evident in internal validation. In the 7:3 stratified split-sample validation, the AUC improved from 0.719 in the baseline model to 0.826 in the combined model, with the Brier score decreasing from 0.214 to 0.167. In 10-fold cross-validation using pooled out-of-fold predictions, the AUC improved from 0.724 to 0.822, and the Brier score decreased from 0.207 to 0.165. Continuous NRI and IDI analyses also supported improved classification after adding regression pattern (**Supplementary Table 9**).

### 3.11 Internal validation

To further assess the internal validity of the prediction model, we performed additional validation using a 7:3 stratified random split and 10-fold cross-validation. In the full cohort, the apparent model performance was AUC 0.875 (95% CI 0.826–0.922), with a Brier score of 0.137. In the 7:3 split-sample analysis, the training AUC was 0.883, whereas the validation AUC was 0.826 (95% CI 0.711–0.924), with a validation Brier score of 0.167. In 10-fold cross-validation, the pooled out-of-fold AUC was 0.822 (95% CI 0.760–0.880), and the corresponding Brier score was 0.165. Calibration plots showed acceptable agreement between predicted and observed risks, and are provided in **Supplementary Figure 8**. These findings support that the model retained moderate-to-good discrimination and acceptable calibration on internal validation, although some performance attenuation was observed compared with the apparent full-cohort results. Detailed discrimination and calibration metrics for the apparent full-cohort model, the 7:3 stratified split-sample validation, and 10-fold cross-validation are summarized in **Supplementary Table 10**.

Additional sensitivity analyses were performed to assess potential confounding by the treatment-to-surgery interval. When the interval was added to the primary multivariable model as a continuous variable, it was not independently associated with ypN positivity (OR 1.03, 95% CI 0.94–1.12, p = 0.516), and the associations between regression pattern and ypN positivity remained materially unchanged (diffuse/mixed vs centripetal: OR 14.59, 95% CI 3.87–54.96; centrifugal vs centripetal: OR 45.94, 95% CI 12.80–164.89). Similar findings were observed when the interval was categorized as <12, 12–16, and >16 weeks: neither the 12–16-week group (OR 0.70, 95% CI 0.29–1.68, p = 0.430) nor the >16-week group (OR 1.55, 95% CI 0.50–4.82, p = 0.454) was independently associated with ypN positivity, while the regression-pattern effect remained stable. Model discrimination and prediction error were also essentially unchanged after interval adjustment (AUC 0.875 and Brier score 0.138 for the continuous-interval model; AUC 0.879 and Brier score 0.137 for the categorical-interval model) (**Supplementary Table 11**).

As an additional descriptive postoperative analysis, LNR differed significantly across regression patterns. In the overall cohort, the median LNR was 0.000 (IQR 0.000–0.000) in centripetal regression, 0.000 (IQR 0.000–0.073) in diffuse/mixed regression, and 0.144 (IQR 0.026–0.277) in centrifugal regression (p < 0.001). Among ypN-positive patients, the corresponding median LNRs were 0.039 (IQR 0.035–0.126), 0.079 (IQR 0.049–0.098), and 0.182 (IQR 0.108–0.327), respectively (p = 0.001), supporting a gradient in postoperative nodal burden across regression patterns (**Supplementary Table 11**).

## 4 Discussion

This study systematically evaluated pathological regression patterns in advanced gastric cancer after neoadjuvant or conversion therapy and confirmed a strong association between these patterns and residual lymph node metastasis, largely independent of histological subtype and baseline clinical factors. Using a simple three-pattern classification (centripetal, diffuse/mixed, centrifugal), we observed a stable gradient in ypN positivity, pathological response, and Becker TRG that persisted after multivariable adjustment and in the ≥16-nodes subgroup. These findings indicate that regression pattern is not a purely descriptive feature but a pathological marker integrating treatment sensitivity and biological aggressiveness.

Previous studies have confirmed that the TRG is a key objective indicator for evaluating the efficacy of NAT in gastric cancer. The lower the proportion of residual tumor, the better the patient’s survival outcome (11, 12). However, subsequent work has shown that relying solely on the TRG is insufficient to explain the differences in prognosis. Incorporating factors such as lymphovascular invasion and lymph node status significantly improves prognostic stratification (13, 14). Later studies attempted to integrate TRG with ypN staging to construct “new staging” systems, and the results demonstrated that ypN status plays a central role in prognostic evaluation (14, 15). Building on this, the focus of this study has advanced further. Instead of merely considering “how much regression” (TRG and residual tumor percentage), we focus on “how the tumor regresses”—the spatial distribution pattern of tumor regression in the primary tumor. For the first time, this study systematically demonstrates the close association between regression patterns and ypN status in a gastric cancer cohort with uniform D2 lymphadenectomy.

It is noteworthy that the concept of tumor regression patterns guiding surgical strategies has begun to emerge in other cancers. For example, in NAT studies for head and neck squamous cell carcinoma and non-small cell lung cancer, large section evaluations found that tumors with concentrated, centripetal-like regression patterns are more likely to achieve MPR. Some studies are exploring whether patients with excellent responses could potentially undergo more limited resections or reduced lymph node dissection (16–18). Similar observations have been made in breast cancer, colorectal cancer, and esophageal cancer, where certain tumors exhibit “multifocal residual” or “non-centripetal regression” distributions, suggesting that if resection is based solely on the size of the reduced tumor, scattered peripheral residual lesions may remain (19–21). Although research on the classification of regression spatial patterns and their relationship with lymph node involvement in gastric cancer is extremely limited, these cross-cancer findings provide background for our hypothesis of “refining surgical approaches and dissection strategies using regression patterns”.

In this study, three regression patterns delineated clearly distinct biological phenotypes. Centripetal regression consistently reflected deep primary tumor response with near-complete nodal clearance, whereas centrifugal regression was associated with substantial residual disease and a heavy nodal burden; diffuse/mixed regression lay between these two extremes. Notably, these gradients emerged despite comparable baseline characteristics, treatment intensity and extent of lymphadenectomy among the three groups, supporting the view that regression pattern primarily captures intrinsic biological response to therapy rather than differences in how patients were treated or operated on.

To move from qualitative impressions to reproducible metrics, we introduced three semi-quantitative indices—PRI, LRI and CER—to characterize residual primary tumor burden, lymph-node regression and the central–peripheral distribution of residual tumor, respectively (15, 22).

These indices differed markedly across regression patterns in a manner that closely mirrored visual assessment (centripetal with near-zero PRI and CER/LRI close to 1, centrifugal showing the opposite, and diffuse/mixed in between), and interobserver agreement for pattern assignment was excellent (median kappa ≈0.90). These findings indicate that regression patterns represent a robust, quantifiable pathological phenotype rather than a purely subjective impression, and provide a methodological basis for future standardization of studies. Representative histological images of the three regression patterns are provided in **Supplementary Figure 9** to facilitate visual standardization and reproducibility. In addition, exploratory restricted cubic spline analyses suggested that the associations of the semi-quantitative indices with ypN positivity were not entirely linear, particularly for PRI and LRI. This finding further supports the interpretation that these indices capture structured pathological gradients related to residual nodal risk, rather than serving merely as descriptive surrogates of visual pattern assignment.

In the multivariable analysis, regression pattern remained the strongest independent predictor of ypN positivity after adjustment for Lauren type, treatment regimen, tumor location, clinical T/N stage, PD-L1 status and total lymph nodes. Compared with centripetal regression, the risk of ypN positivity increased approximately 14-fold for diffuse/mixed regression and 43-fold for centrifugal regression, whereas clinical N stage exerted only 1.8-fold independent effect. In the subgroup with ≥16 lymph nodes, this effect remained nearly unchanged, indicating that the observed association was not a “spurious effect caused by inadequate lymph node dissection.” This finding aligns with the consensus in previous studies, which state that “at least 16 lymph nodes should be retrieved to ensure accurate staging” (23, 24). *Post-hoc* contrasts further showed that, among non-centripetal cases, centrifugal regression carried roughly three times the ypN risk of diffuse/mixed regression, while no meaningful difference was observed between Lauren diffuse and mixed types, even in the LN≥16 subgroup. This finding suggests that the regression pattern is not simply a “replica” of Lauren classification but reflects treatment-induced changes in tumor tissue structure and microenvironment remodeling (25).

We also observed a distinct distribution of PD-L1 expression across regression patterns, with CPS ≥5 being more frequent in the centripetal group, consistent with immunologic studies suggesting that “inflammatory” microenvironments are more likely to achieve deep regression (26).

However, PD-L1 was not an independent predictor of ypN status in multivariable analyses, whereas the effect of regression pattern remained robust. This supports the view that regression pattern functions as a composite pathological index integrating multiple biological signals, rather than a surrogate for any single molecular marker.

The logistic regression model demonstrated good discriminatory ability and calibration in both the full cohort and the subgroup with ≥16 lymph nodes. The marginal predicted risks showed a clear stepwise increase in ypN positivity from centripetal to diffuse/mixed to centrifugal regression, whereas Lauren classification clustered within a narrower and largely overlapping risk range. This contrast underscores the incremental value of regression patterns for lymph node risk stratification and supports their incorporation into future multidimensional prediction models. Nevertheless, the present model should be regarded as a proof-of-concept tool rather than a ready-to-use decision aid, and its clinical utility still requires external validation and prospective evaluation. Moreover, this incremental value was further supported by additional sensitivity analyses. The association between regression pattern and ypN positivity persisted after further adjustment for conventional post-treatment primary tumor response measures, including ypT and Becker TRG, and also remained evident in the non-pCR subgroup. These findings suggest that regression pattern is not merely a surrogate for response depth, but captures additional spatial-pathological information relevant to residual nodal risk. Importantly, this incremental value was also evident when regression pattern was added to a baseline model composed of conventional clinicopathological predictors available in our dataset. Compared with the baseline model, the combined model showed improved discrimination and reduced prediction error in both the full cohort and internal validation analyses, supporting that regression pattern provides information beyond established predictors rather than merely recapitulating them.

Although additional internal validation using split-sample analysis and 10-fold cross-validation showed that model discrimination and calibration remained acceptable, this study remains a single-center analysis without external validation. Therefore, the current model should be interpreted as an internally assessed proof-of-concept tool rather than a ready-to-use clinical decision aid, and independent validation in multicenter cohorts is still required before clinical implementation.

Importantly, because regression patterns were defined from postoperative resected specimens, their current value should be interpreted primarily in the postoperative setting, including nodal-risk assessment, prognostic stratification, postoperative multidisciplinary discussion, and surveillance planning. Their potential application to individualized lymphadenectomy should be regarded as a future translational direction rather than a current practice recommendation. In current surgical practice, D2 lymphadenectomy remains the recommended standard for resectable advanced gastric cancer (4, 5, 23). However, questions remain regarding how best to manage patients with persistently high nodal-risk features after neoadjuvant therapy, including whether more intensive locoregional strategies may be relevant in selected settings (27). In our cohort, all patients underwent standard D2 lymphadenectomy, which minimized confounding related to variation in the extent of nodal dissection. Accordingly, our findings should not be interpreted as supporting real-time modification of lymphadenectomy extent before or during the same operation. Rather, the immediate clinical value of regression patterns lies in postoperative pathological risk stratification. Centripetal regression may identify a very low residual nodal-risk phenotype, whereas centrifugal regression may identify patients with persistently high nodal burden who may warrant closer surveillance, more intensive postoperative multidisciplinary discussion, and more cautious prognostic assessment. At the same time, these findings may still have translational implications. Specifically, regression patterns may serve as a biological reference framework for future efforts to develop validated preoperative or intraoperative surrogate tools that could eventually support more individualized lymphadenectomy strategies in selected patients. However, this pathway remains hypothesis-generating and requires prospective validation before any change in surgical practice can be considered.

Additional sensitivity analyses showed that inclusion of the treatment-to-surgery interval, either as a continuous variable or in prespecified categories, did not materially alter the association between regression pattern and ypN positivity, arguing against major confounding by treatment timing in this cohort. As an additional supportive postoperative analysis, LNR also differed significantly across regression patterns, with the highest nodal burden observed in centrifugal regression. Although LNR is a well-established postoperative nodal-burden metric, it was not treated as a confounder in the primary model because it is derived from postoperative nodal status and therefore lies downstream of the ypN outcome. Instead, it was analyzed descriptively as a supportive postoperative indicator.

This study has several limitations. First, it is a single-center retrospective analysis with a moderate sample size, so selection bias cannot be excluded and external generalizability is limited; the inclusion of a small number of limited cM1 “conversion therapy” cases does not fully match the strict inclusion criteria of some prospective trials but is more reflective of real-world clinical practice. Second, regression patterns were assessed by experienced gastrointestinal pathologists under relatively standardized sampling, and the reproducibility of this classification in other centers—especially without large or standardized sections—requires multicenter validation.

Third, we focused on ypN ≥1 as the primary endpoint, addressing the clinically relevant question of “any residual nodal metastasis,” but did not analyze more granular metrics such as nodal station involvement and lymph node regression grading. Fourth, neoadjuvant/conversion regimens were heterogeneous (from chemo to chemo+IO+targeted), which reflects current practice but limits detailed comparison of regimen-specific effects. Finally, survival outcomes were not available; whether regression patterns provide incremental prognostic stratification needs to be clarified in future studies with adequate follow-up. An additional limitation is that regression patterns were defined from postoperative resected specimens; therefore, their current utility is mainly postoperative, and they cannot directly support real-time tailoring of lymphadenectomy extent without validated preoperative or intraoperative surrogate markers.

In the future, if regression patterns are validated in independent external cohorts and further shown to be closely related to survival outcomes, they could be integrated into comprehensive risk assessment models for NAT to help optimize the indications for lymphadenectomy.

## 5 Conclusion

This study shows that pathological regression patterns (centripetal, diffuse/mixed, and centrifugal) after neoadjuvant or conversion therapy in gastric cancer are strongly associated with residual lymph node metastasis and form a clear stepwise gradient of ypN risk. Supported by quantitative indices (PRI, LRI, and CER), regression patterns represent a simple, reproducible, and quantifiable pathological phenotype that provides additional nodal-risk stratification value beyond traditional clinicopathologic factors. At present, their clinical utility is best interpreted in the postoperative setting, particularly for nodal-risk assessment, prognostic stratification, postoperative multidisciplinary discussion, and surveillance planning. If validated in multicenter cohorts with survival data, regression patterns could be integrated with TRG and ypN into post-neoadjuvant risk assessment systems. Their potential application to individualized lymphadenectomy should be regarded as a future translational direction that would require validated preoperative or intraoperative surrogate markers and prospective confirmation.

## Data Availability

The original contributions presented in the study are included in the article/**Supplementary Material**. Further inquiries can be directed to the corresponding author.
