# Supplementary material for: From centripetal to centrifugal: pathological regression patterns after neoadjuvant or conversion therapy as markers of nodal risk and a framework for future research on individualized lymphadenectomy in gastric cancer
Source: Front Immunol. 2026 Apr 13;17:1766242. doi: 10.3389/fimmu.2026.1766242 (PMC13111328; doi:10.3389/fimmu.2026.1766242)
Supplement: Supplementary file 1 [file Supplementaryfile1.docx]

**Supplementary Table 1. Baseline clinicopathologic and treatment characteristics of the study cohort (n = 195)**

| Category | Variable | | Overall (n = 195) |
| --- | --- | --- | --- |
| Demographics | Age, years (median, IQR) | | 59.000 (54.000,66.000) |
|  | BMI, kg/m² (median, IQR) | | 23.100 (22.300,24.100) |
|  | Sex, n (%) | Female | 26 (13.33) |
|  |  | Male | 169 (86.67) |
|  | ECOG status, n (%) | 0 | 17 (8.72) |
|  |  | 1 | 163 (83.59) |
|  |  | 2 | 15 (7.69) |
| Tumor characteristics | Tumor location, n (%) | body | 58 (29.74) |
|  |  | antrum | 67 (34.36) |
|  |  | cardia | 70 (35.90) |
|  | Lauren classification, n (%) | diffuse | 71 (36.41) |
|  |  | mixed | 28 (14.36) |
|  |  | intestinal | 96 (49.23) |
|  | Histologic grade, n (%) | Moderate-Low Grade | 111 (56.92) |
|  |  | Moderate Grade | 5 (2.57) |
|  |  | Low Grade | 79 (40.51) |
|  | Special histologic subtype, n (%) | EBV Infection | 3 (23.08) |
|  |  | Signet Ring Cell Carcinoma | 3 (23.08) |
|  |  | Neuroendocrine | 3 (23.08) |
|  |  | Mucinous Adenocarcinoma | 4 (30.76) |
|  | Borrmann type, n (%) | II | 12 (6.15) |
|  |  | III | 165 (84.62) |
|  |  | IV | 18 (9.23) |
|  | Clinical T stage, n (%) | 2 | 7 (3.60) |
|  |  | 3 | 94 (48.20) |
|  |  | 4 | 94 (48.20) |
|  | Clinical N stage, n (%) | 0 | 15 (7.69) |
|  |  | 1 | 45 (23.08) |
|  |  | 2 | 89 (45.64) |
|  |  | 3 | 46 (23.59) |
|  | Clinical M stage, n (%) | 0 | 178 (91.28) |
|  |  | 1 | 17 (8.72) |
| Treatment characteristics | Treatment regimen, n (%) | chemo | 47 (24.10) |
|  |  | chemo+IO | 100 (51.28) |
|  |  | chemo+IO+targeted | 48 (24.62) |
|  | preoperative cycles (median, IQR) | | 4.000 (3.000,4.000) |
|  | Interval from NAT start to surgery, weeks (median, IQR) | | 14.000 (11.000,16.000) |
| Biomarkers | HER2 status, n (%) | 0 | 148 (75.90) |
|  |  | 1+ | 22 (11.28) |
|  |  | 2+ | 10 (5.13) |
|  |  | 3+ | 13 (6.67) |
|  |  | 4+ | 2 (1.02) |
|  | MSI status, n (%) | MSI-H | 7 (3.59) |
|  |  | MSS | 188 (96.41) |
|  | PD-L1 status, n (%) | CPS<5 | 113 (57.95) |
|  |  | CPS≥5 | 82 (42.05) |
| Surgical characteristics | Type of gastrectomy, n (%) | total | 102 (52.31) |
|  |  | distal | 67 (34.36) |
|  |  | proximal | 26 (13.33) |
|  | Resection margin status, n (%) | R0 | 194 (99.49) |
|  |  | R1 | 1 (0.51) |
|  | Length of hospital stay, days (median, IQR) | | 14.000 (12.000,17.000) |

Data are presented as n (%) unless otherwise specified. Continuous variables are summarized as median (interquartile range, IQR).

Abbreviations: BMI, body mass index; ECOG, Eastern Cooperative Oncology Group; NAT, neoadjuvant/conversion therapy; chemo, chemotherapy; IO, immunotherapy; HER2, human epidermal growth factor receptor 2; MSI, microsatellite instability; MSS, microsatellite stable; PD-L1, programmed death-ligand 1; CPS, combined positive score; EBV, Epstein–Barr virus.

**Supplementary Table 2. Univariable logistic regression analyses of candidate predictors of ypN positivity**

| Variable | OR | 95% CI | p |
| --- | --- | --- | --- |
| Regression: Diffuse/Mixed vs Centripetal | 15.22 | 4.71 ~ 49.14 | 0.000** |
| Regression: Centrifugal vs Centripetal | 54.34 | 17.51 ~ 168.64 | 0.000** |
| Lauren: Diffuse vs Intestinal | 6.99 | 3.50 ~ 13.98 | 0.000** |
| Lauren: Mixed vs Intestinal | 5.87 | 2.38 ~ 14.51 | 0.000** |
| Regimen: chemo+IO vs chemo | 0.97 | 0.48 ~ 1.94 | 0.927 |
| Regimen: chemo+IO+targeted vs chemo | 0.52 | 0.22 ~ 1.19 | 0.122 |
| Location: Antrum vs Body | 0.68 | 0.33 ~ 1.37 | 0.278 |
| Location: Cardia vs Body | 0.63 | 0.31 ~ 1.27 | 0.196 |
| cT | 1.60 | 0.95 ~ 2.67 | 0.076 |
| cN | 1.42 | 1.01 ~ 2.00 | 0.041* |
| PD-L1 | 0.55 | 0.30 ~ 0.98 | 0.044* |
| Total LN Count | 1.00 | 0.97 ~ 1.03 | 0.929 |

Abbreviations: OR, odds ratio; CI, confidence interval; LN, lymph node; chemo, chemotherapy; IO, immunotherapy. OR > 1 indicates increased odds of ypN positivity compared with the reference category. *p < 0.05, **p < 0.01.

**Supplementary Table 3. Multivariable logistic regression analysis of predictors of ypN positivity in patients with ≥16 examined lymph nodes**

| Variable | OR | 95% CI | p |
| --- | --- | --- | --- |
| Regression: Diffuse/Mixed vs Centripetal | 13.092 | 3.395 ~ 50.492 | 0.000** |
| Regression: Centrifugal vs Centripetal | 43.63 | 11.56 ~ 164.674 | 0.000** |
| Lauren: Diffuse vs Intestinal | 1.902 | 0.677 ~ 5.341 | 0.222 |
| Lauren: Mixed vs Intestinal | 2.091 | 0.585 ~ 7.469 | 0.256 |
| Regimen: chemo+IO vs chemo | 1.875 | 0.611 ~ 5.752 | 0.272 |
| Regimen: chemo+IO+targeted vs chemo | 1.372 | 0.361 ~ 5.21 | 0.643 |
| Location: Antrum vs Body | 1.06 | 0.343 ~ 3.277 | 0.919 |
| Location: Cardia vs Body | 0.578 | 0.2 ~ 1.672 | 0.312 |
| cT | 0.962 | 0.411 ~ 2.249 | 0.928 |
| cN | 2.112 | 1.193 ~ 3.74 | 0.012* |
| PD-L1 | 0.564 | 0.22 ~ 1.45 | 0.235 |
| Total LN Count | 0.958 | 0.898 ~ 1.022 | 0.191 |

Abbreviations: OR, odds ratio; CI, confidence interval; LN, lymph node; chemo, chemotherapy; IO, immunotherapy. This model was restricted to patients with ≥16 examined lymph nodes. OR > 1 indicates increased odds of ypN positivity compared with the reference category. *p < 0.05, **p < 0.01.

**Supplementary Table 4. Multivariable logistic regression analysis of predictors of ypN positivity after excluding cT**

| Variable | OR | 95% CI | p |
| --- | --- | --- | --- |
| Regression: Diffuse/Mixed vs Centripetal | 13.966 | 3.760 ~ 51.870 | 0.000** |
| Regression: Centrifugal vs Centripetal | 43.447 | 12.355 ~ 152.776 | 0.000** |
| Lauren: Diffuse vs Intestinal | 2.142 | 0.843 ~ 5.446 | 0.109 |
| Lauren: Mixed vs Intestinal | 2.158 | 0.671 ~ 6.938 | 0.197 |
| Regimen: chemo+IO vs chemo | 1.432 | 0.541 ~ 3.788 | 0.470 |
| Regimen: chemo+IO+targeted vs chemo | 1.046 | 0.320 ~ 3.419 | 0.940 |
| Location: Antrum vs Body | 1.146 | 0.403 ~ 3.258 | 0.798 |
| Location: Cardia vs Body | 0.599 | 0.227 ~ 1.580 | 0.300 |
| cN | 1.795 | 1.113 ~ 2.894 | 0.016* |
| PD-L1 | 0.752 | 0.317 ~ 1.782 | 0.517 |
| Total LN Count | 0.978 | 0.931 ~ 1.028 | 0.389 |

Model performance: AUC = 0.875; Hosmer–Lemeshow p = 0.304. Abbreviations: OR, odds ratio; CI, confidence interval; LN, lymph node; chemo, chemotherapy; IO, immunotherapy. *p < 0.05, **p < 0.01.

**Supplementary Table 5. Multivariable logistic regression analysis of predictors of ypN positivity with simplified binary coding of cT and cN**

| Variable | OR | 95% CI | p |
| --- | --- | --- | --- |
| Regression: Diffuse/Mixed vs Centripetal | 12.698 | 3.498 ~ 46.091 | 0.000** |
| Regression: Centrifugal vs Centripetal | 37.808 | 11.069 ~ 129.145 | 0.000** |
| Lauren: Diffuse vs Intestinal | 2.115 | 0.824 ~ 5.433 | 0.119 |
| Lauren: Mixed vs Intestinal | 2.051 | 0.650 ~ 6.478 | 0.221 |
| Regimen: chemo+IO vs chemo | 1.555 | 0.579 ~ 4.178 | 0.381 |
| Regimen: chemo+IO+targeted vs chemo | 1.105 | 0.342 ~ 3.569 | 0.867 |
| Location: Antrum vs Body | 1.209 | 0.432 ~ 3.381 | 0.718 |
| Location: Cardia vs Body | 0.633 | 0.238 ~ 1.680 | 0.358 |
| cT: cT4 vs cT2–3 | 1.310 | 0.579 ~ 2.962 | 0.516 |
| cN: cN2–3 vs cN0–1 | 1.746 | 0.742 ~ 4.112 | 0.202 |
| PD-L1 | 0.803 | 0.345 ~ 1.871 | 0.611 |
| Total LN Count | 0.985 | 0.938 ~ 1.034 | 0.539 |

Model performance: AUC = 0.869; Hosmer–Lemeshow p = 0.105. Abbreviations: OR, odds ratio; CI, confidence interval; LN, lymph node; chemo, chemotherapy; IO, immunotherapy. **p < 0.01.

**Supplementary Table 6. Multivariable logistic regression analysis of predictors of ypN positivity in the overall cohort, with additional adjustment for ypT**

| Variable | OR | 95% CI | p |
| --- | --- | --- | --- |
| Regression: Diffuse/Mixed vs Centripetal | 7.414 | 1.821 ~ 30.191 | 0.005* |
| Regression: Centrifugal vs Centripetal | 17.863 | 4.391 ~ 72.662 | 0.000** |
| Lauren: Diffuse vs Intestinal | 1.587 | 0.600 ~ 4.199 | 0.353 |
| Lauren: Mixed vs Intestinal | 1.662 | 0.507 ~ 5.454 | 0.402 |
| Regimen: chemo+IO vs chemo | 1.701 | 0.613 ~ 4.720 | 0.308 |
| Regimen: chemo+IO+targeted vs chemo | 0.997 | 0.299 ~ 3.329 | 0.996 |
| Location: Antrum vs Body | 1.267 | 0.441 ~ 3.640 | 0.661 |
| Location: Cardia vs Body | 0.589 | 0.221 ~ 1.572 | 0.291 |
| cT | 0.951 | 0.434 ~ 2.081 | 0.900 |
| cN | 1.696 | 1.026 ~ 2.802 | 0.039* |
| PD-L1 | 0.728 | 0.303 ~ 1.748 | 0.477 |
| Total LN Count | 0.973 | 0.924 ~ 1.024 | 0.290 |
| ypT | 1.766 | 1.120 ~ 2.784 | 0.014* |

Abbreviations: OR, odds ratio; CI, confidence interval; LN, lymph node; chemo, chemotherapy; IO, immunotherapy. OR > 1 indicates increased odds of ypN positivity compared with the reference category. *p < 0.05, **p < 0.01.

**Supplementary Table 7. Multivariable logistic regression analysis of predictors of ypN positivity in the overall cohort, with additional adjustment for Becker TRG**

| Variable | OR | 95% CI | p |
| --- | --- | --- | --- |
| Regression: Diffuse/Mixed vs Centripetal | 15.477 | 3.501 ~ 68.421 | 0.000** |
| Regression: Centrifugal vs Centripetal | 55.053 | 7.113 ~ 426.108 | 0.000** |
| Lauren: Diffuse vs Intestinal | 2.205 | 0.821 ~ 5.919 | 0.117 |
| Lauren: Mixed vs Intestinal | 2.225 | 0.679 ~ 7.286 | 0.186 |
| Regimen: chemo+IO vs chemo | 1.445 | 0.535 ~ 3.903 | 0.468 |
| Regimen: chemo+IO+targeted vs chemo | 1.046 | 0.318 ~ 3.441 | 0.940 |
| Location: Antrum vs Body | 1.134 | 0.397 ~ 3.244 | 0.814 |
| Location: Cardia vs Body | 0.602 | 0.227 ~ 1.593 | 0.306 |
| cT | 1.035 | 0.479 ~ 2.234 | 0.931 |
| cN | 1.777 | 1.089 ~ 2.900 | 0.021* |
| PD-L1 | 0.739 | 0.310 ~ 1.763 | 0.495 |
| Total LN Count | 0.978 | 0.930 ~ 1.028 | 0.387 |
| Becker TRG | 0.860 | 0.311 ~ 2.377 | 0.771 |

Abbreviations: OR, odds ratio; CI, confidence interval; LN, lymph node; TRG, tumor regression grade; chemo, chemotherapy; IO, immunotherapy. OR > 1 indicates increased odds of ypN positivity compared with the reference category. *p < 0.05, **p < 0.01.

**Supplementary Table 8. Multivariable logistic regression analysis of predictors of ypN positivity in the non-pCR subgroup (n = 155)**

| Variable | OR | 95% CI | p |
| --- | --- | --- | --- |
| Regression: Diffuse/Mixed vs Centripetal | 7.599 | 2.018 ~ 28.618 | 0.003* |
| Regression: Centrifugal vs Centripetal | 22.993 | 6.459 ~ 81.845 | 0.000** |
| Lauren: Diffuse vs Intestinal | 1.757 | 0.687 ~ 4.498 | 0.240 |
| Lauren: Mixed vs Intestinal | 1.832 | 0.579 ~ 5.803 | 0.303 |
| Regimen: chemo+IO vs chemo | 1.514 | 0.567 ~ 4.041 | 0.408 |
| Regimen: chemo+IO+targeted vs chemo | 1.085 | 0.332 ~ 3.546 | 0.893 |
| Location: Antrum vs Body | 1.148 | 0.402 ~ 3.278 | 0.796 |
| Location: Cardia vs Body | 0.567 | 0.214 ~ 1.505 | 0.255 |
| cT | 1.099 | 0.506 ~ 2.386 | 0.812 |
| cN | 1.722 | 1.034 ~ 2.867 | 0.037* |
| PD-L1 | 0.758 | 0.319 ~ 1.803 | 0.531 |
| Total LN Count | 0.973 | 0.925 ~ 1.023 | 0.282 |

Abbreviations: OR, odds ratio; CI, confidence interval; pCR, pathological complete response; LN, lymph node; chemo, chemotherapy; IO, immunotherapy. OR > 1 indicates increased odds of ypN positivity compared with the reference category. *p < 0.05, **p < 0.01.

**Supplementary Table 9. Incremental predictive value of regression pattern beyond conventional clinicopathological predictors for ypN positivity.**

| Model setting | AUC | Brier score | LR test p value | Continuous NRI | IDI |
| --- | --- | --- | --- | --- | --- |
| Baseline model (full cohort) | 0.777 | 0.188 | - | - | - |
| Combined model (full cohort) | 0.875 | 0.137 | 2.45 × 10⁻¹² | 1.090 | 0.211 |
| Baseline model (7:3 validation) | 0.719 | 0.214 | - | - | - |
| Combined model (7:3 validation) | 0.826 | 0.167 | - | - | - |
| Baseline model (10-fold CV) | 0.724 | 0.207 | - | - | - |
| Combined model (10-fold CV) | 0.822 | 0.165 | - | 1.079 | 0.201 |

**Supplementary Table 10. Discrimination and calibration performance of the multivariable logistic regression model for ypN positivity in the apparent full-cohort model and internal validation analyses.**

| Validation setting | AUC (95% CI) | Brier score | Calibration assessment |
| --- | --- | --- | --- |
| Apparent full-cohort model | 0.875 (0.826 ~ 0.922) | 0.137 | Acceptable calibration; calibration plot shown in Supplementary Figure 8 |
| 7:3 stratified split-sample validation set | 0.826 (0.711 ~ 0.924) | 0.167 | Acceptable calibration with mild attenuation compared with the apparent model; calibration plot shown in Supplementary Figure 8 |
| 10-fold cross-validation (pooled out-of-fold predictions) | 0.822 (0.760 ~ 0.880) | 0.165 | Acceptable calibration with mild attenuation compared with the apparent model; calibration plot shown in Supplementary Figure 8 |

Abbreviations: AUC, area under the receiver operating characteristic curve; CI, confidence interval. Calibration plots for the apparent full-cohort model, the 7:3 stratified split-sample validation, and 10-fold cross-validation are presented in Supplementary Figure 8.

**Supplementary Table 11. Sensitivity analyses for treatment-to-surgery interval and descriptive postoperative lymph node ratio across regression patterns.**

**Panel A. Sensitivity analyses including treatment-to-surgery interval**

| Model | Interval term | OR (95% CI) | p | Diffuse/mixed vs centripetal OR (95% CI) | Centrifugal vs centripetal OR  (95% CI) | AUC | Brier score |
| --- | --- | --- | --- | --- | --- | --- | --- |
| Primary model | - | - | - | 13.96 (3.75 ~ 51.90) | 43.40 (12.33 ~ 152.78) | 0.875 | 0.137 |
| Primary model + interval (continuous) | Per 1-week increase | 1.03 (0.94 ~ 1.12) | 0.516 | 14.59 (3.87 ~ 54.96) | 45.94 (12.80 ~ 164.89) | 0.875 | 0.138 |
| Primary model + interval (categorical) | 12–16 vs <12 weeks | 0.70 (0.29 ~ 1.68) | 0.430 | 13.78 (3.68 ~ 51.62) | 43.34 (12.25 ~ 153.29) | 0.879 | 0.137 |
| Primary model + interval (categorical) | >16 vs <12 weeks | 1.55 (0.50 ~ 4.82) | 0.454 | 13.78 (3.68 ~ 51.62) | 43.34 (12.25 ~ 153.29) | 0.879 | 0.137 |

**Panel B. Postoperative lymph node ratio across regression patterns**

| Analysis set | Centripetal regression, median (IQR) | Diffuse/mixed regression, median (IQR) | Centrifugal regression, median (IQR) | p |
| --- | --- | --- | --- | --- |
| Overall cohort | 0.000 (0.000 ~ 0.000) | 0.000 (0.000 ~ 0.073) | 0.144 (0.026 ~ 0.277) | <0.001 |
| ypN-positive subgroup | 0.039 (0.035 ~ 0.126) | 0.079 (0.049 ~ 0.098) | 0.182 (0.108 ~ 0.327) | 0.001 |

Abbreviations: AUC, area under the receiver operating characteristic curve; CI, confidence interval; IQR, interquartile range; LNR, lymph node ratio; OR, odds ratio. The primary model included regression pattern, cT, cN, Lauren classification, tumor location, treatment regimen, PD-L1 status, and total retrieved lymph node count. p values for Panel B were calculated using the Kruskal–Wallis test.
