# Supplementary material for: From centripetal to centrifugal: pathological regression patterns after neoadjuvant or conversion therapy as markers of nodal risk and a framework for future research on individualized lymphadenectomy in gastric cancer
Source: Front Immunol. 2026 Apr 13;17:1766242. doi: 10.3389/fimmu.2026.1766242 (PMC13111328; doi:10.3389/fimmu.2026.1766242)
Supplement: Supplementary file 2 [file Supplementaryfile2.docx]

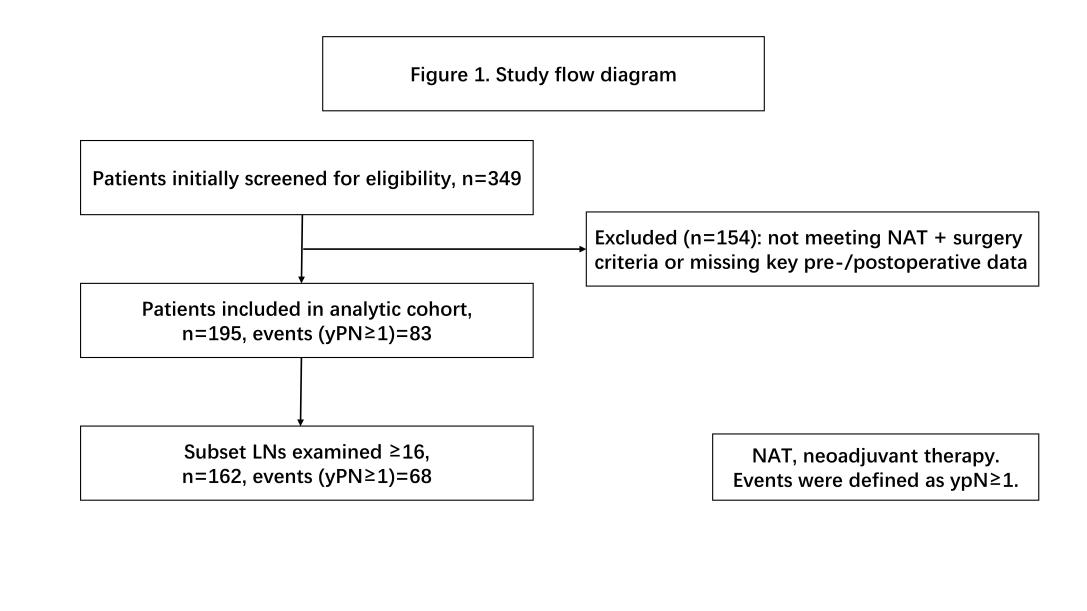


**Supplementary Figure 1. Study flow diagram of patient selection and analytic cohorts**


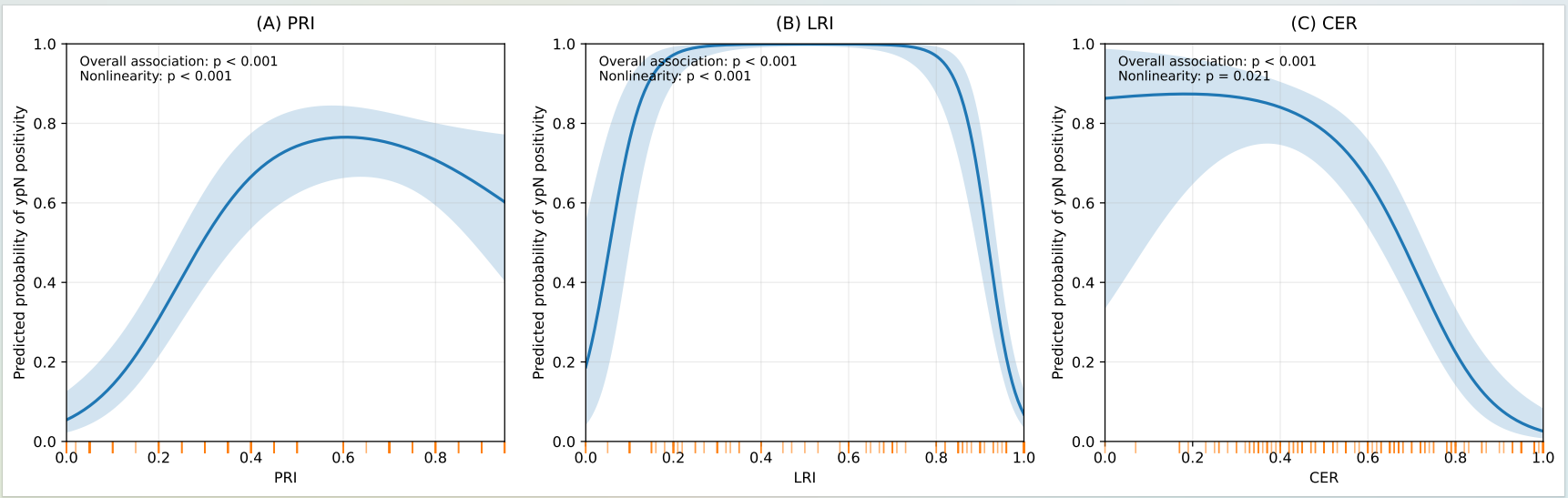


**Supplementary Figure 2. Restricted cubic spline curves for the associations of PRI, LRI, and CER with ypN positivity.** Restricted cubic spline analyses were performed using a parsimonious spline specification to explore the associations between the semi-quantitative regression indices and ypN positivity. Panels show the estimated probability of ypN positivity across the range of PRI (A), LRI (B), and CER (C), with shaded areas indicating 95% confidence intervals. Rug marks along the x-axis indicate the distribution of observed values. PRI and LRI showed significant nonlinear associations with ypN positivity, whereas CER showed a significant overall association with a weaker nonlinear component.


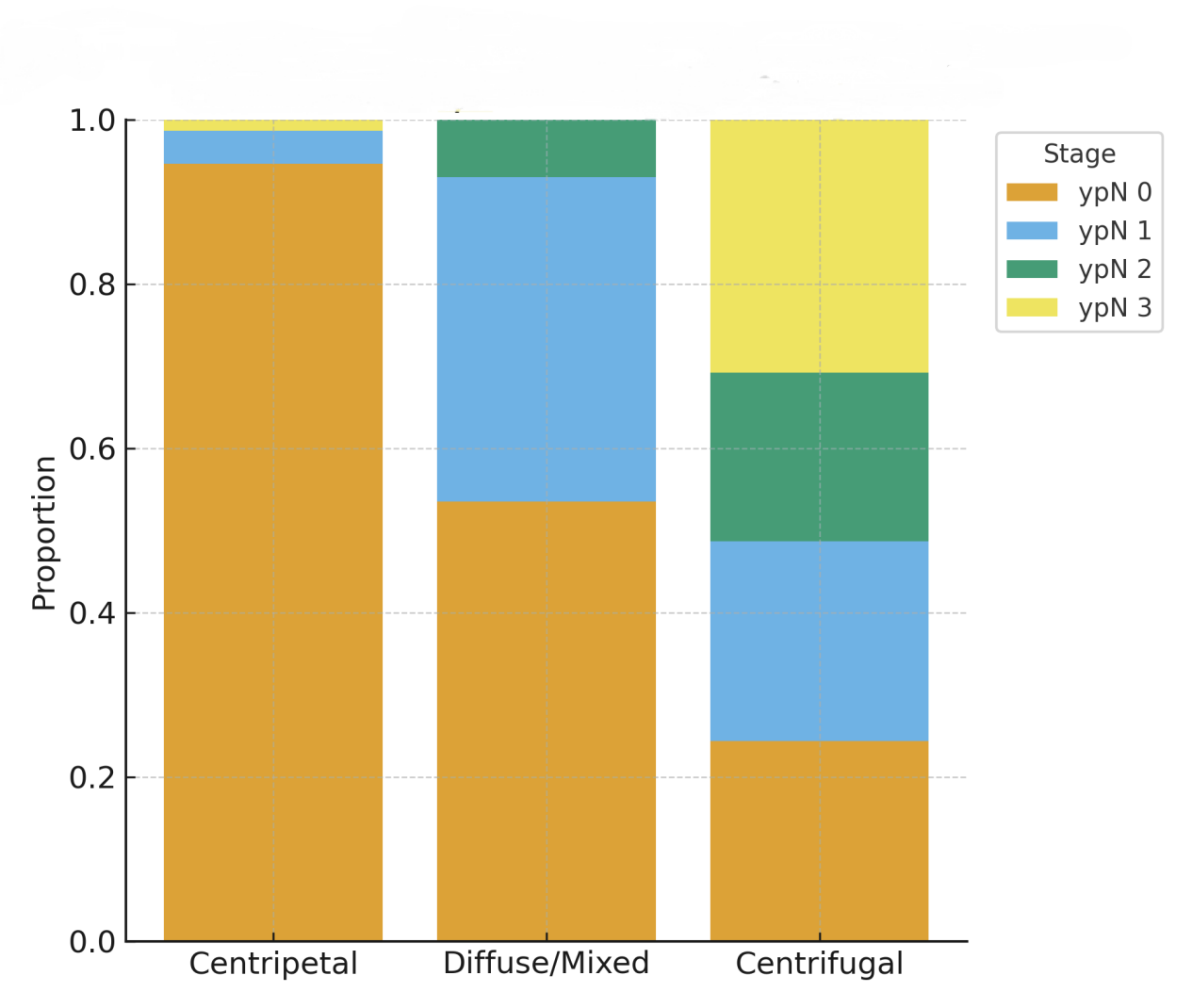


**Supplementary Figure 3. Distribution of ypN stage according to three regression patterns**


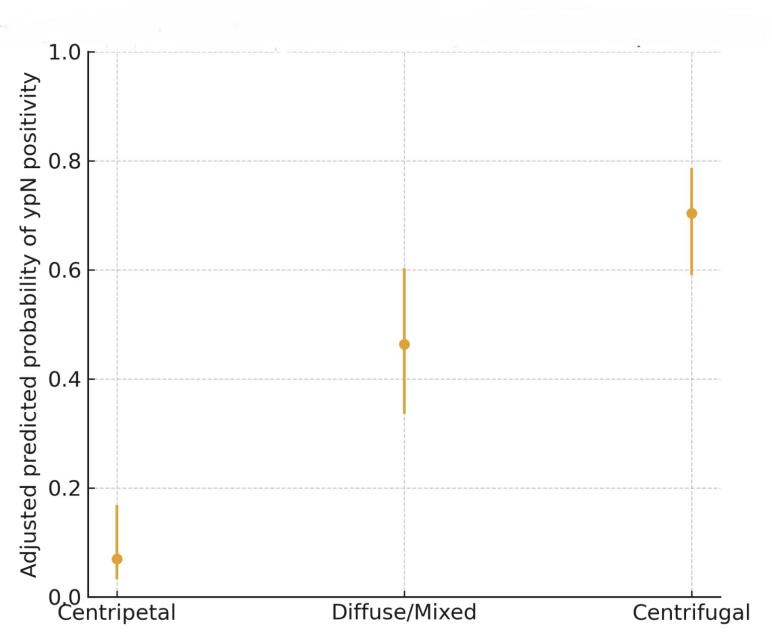


**Supplementary Figure 4. Adjusted predicted probability of ypN positivity according to regression pattern (centripetal, diffuse/mixed, centrifugal) based on the main multivariable model (95% confidence intervals)**


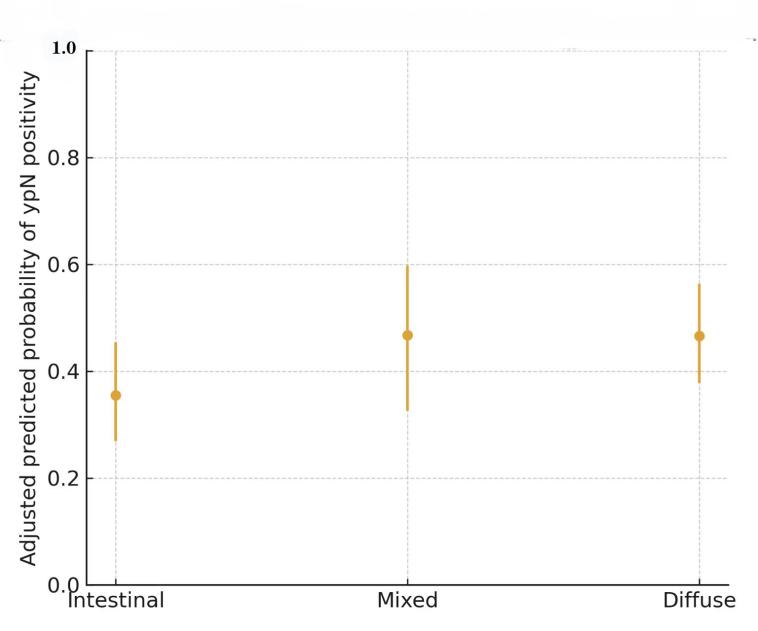


**Supplementary Figure 5. Adjusted predicted probability of ypN positivity according to Lauren classification (intestinal, mixed, diffuse) based on the main multivariable model (95% confidence intervals)**


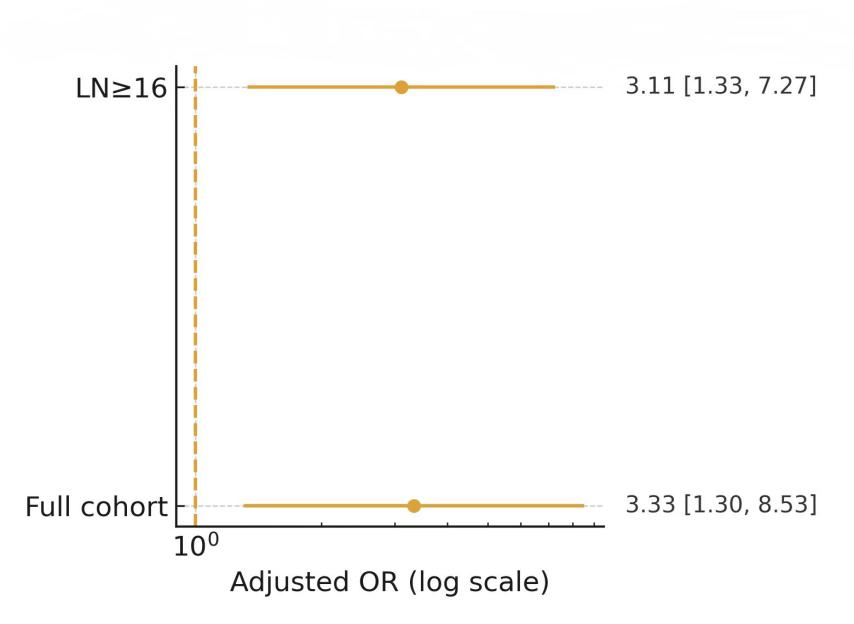


**Supplementary Figure 6. Pairwise adjusted odds ratios for ypN positivity comparing centrifugal vs diffuse/mixed regression patterns in the full cohort and in patients with ≥16 lymph nodes**


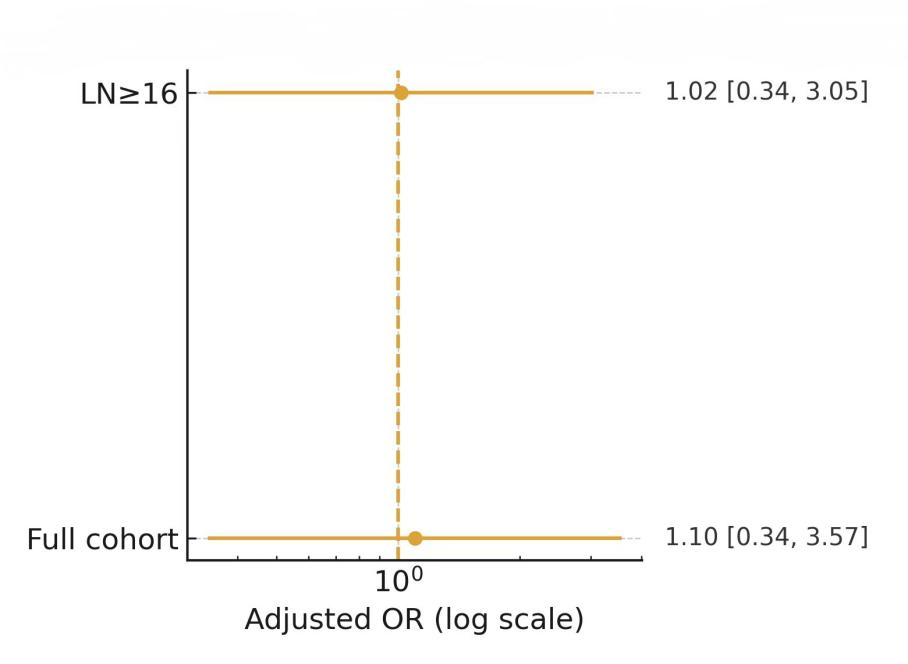


**Supplementary Figure 7. Pairwise adjusted odds ratios for ypN positivity comparing Lauren mixed vs diffuse types in the full cohort and in patients with ≥16 lymph nodes**


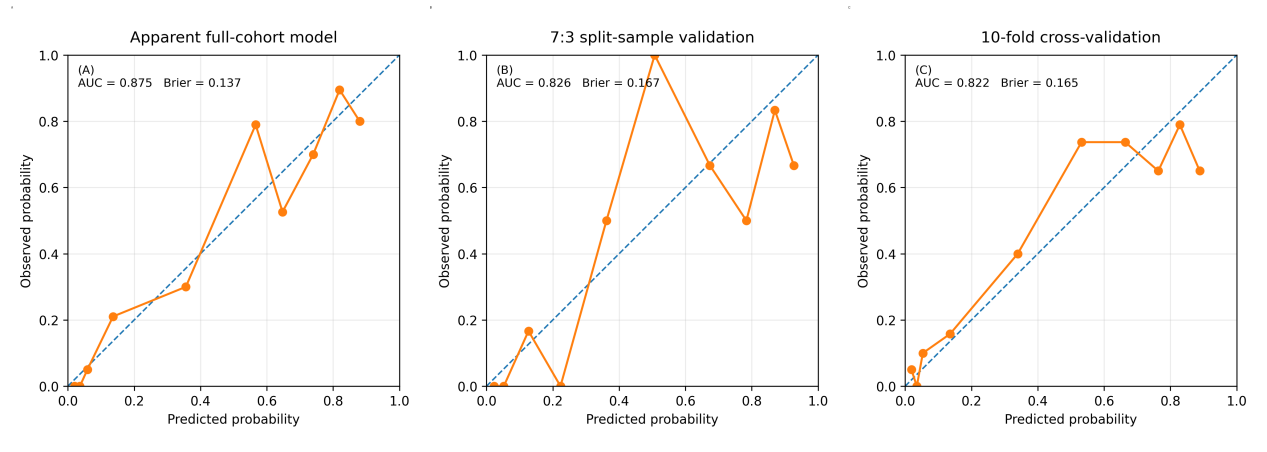


**Supplementary Figure 8. Calibration plots of the multivariable logistic regression model for ypN positivity.**

**Supplementary Figure 9. Representative histological images of the three tumor regression patterns.** All images were obtained from postoperative resected specimens and are provided to illustrate the morphologic basis of regression-pattern classification and to improve reproducibility of pathological assessment.

| **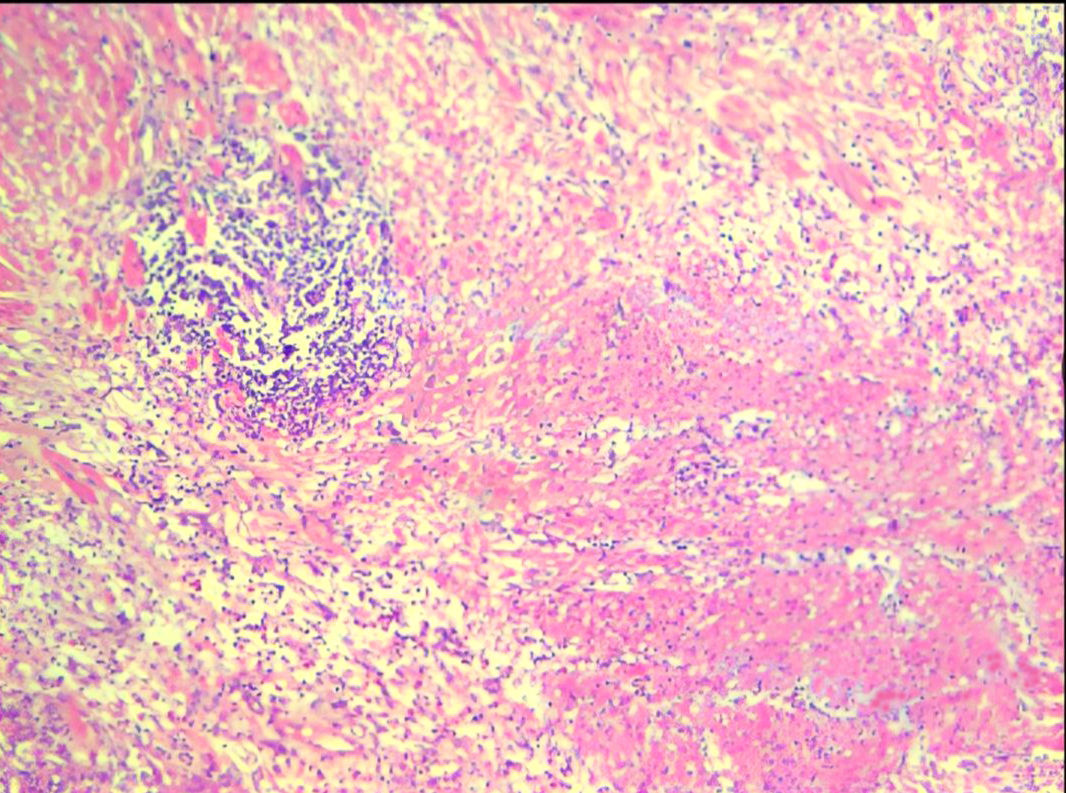**  **A** | **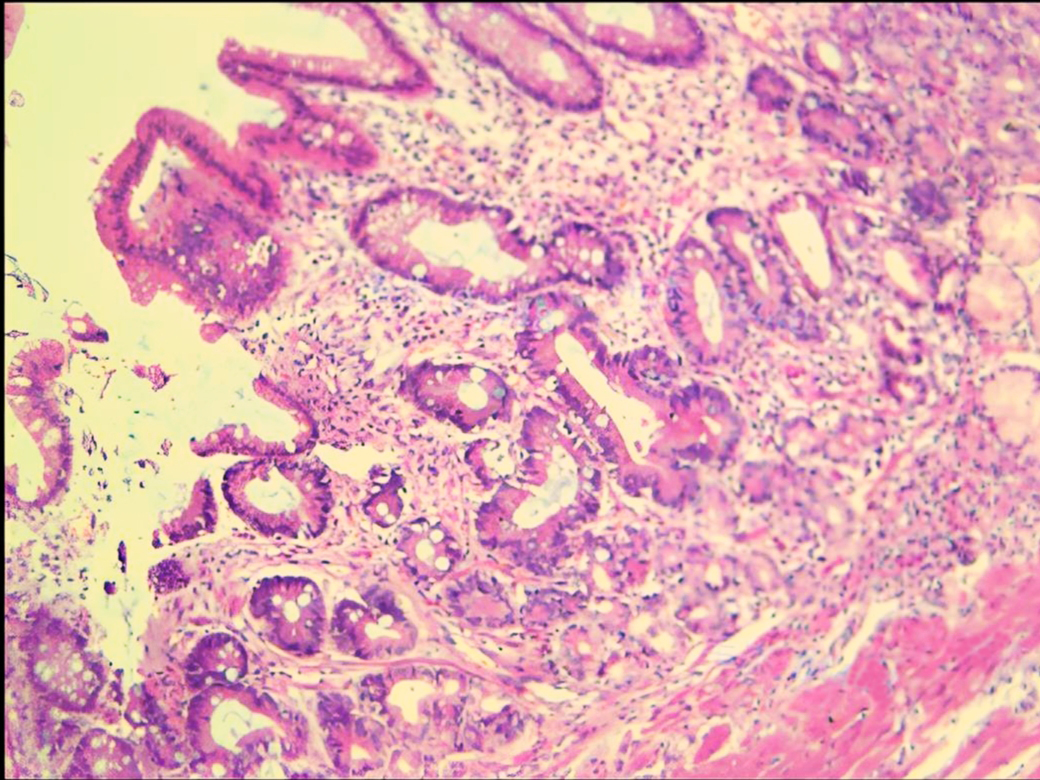**  **B** |
| --- | --- |

(A, B) Centripetal regression pattern. At low magnification (A), the tumor bed is predominantly composed of extensive fibrosis and treatment-related stromal regression, with residual viable tumor appearing relatively localized rather than diffusely distributed. At higher magnification (B), residual adenocarcinoma glands are identified within the regressive stroma, confirming the presence of viable tumor cells in a background of marked treatment effect. This pattern is characterized by a relatively concentrated residual tumor focus with surrounding regressive change.

| 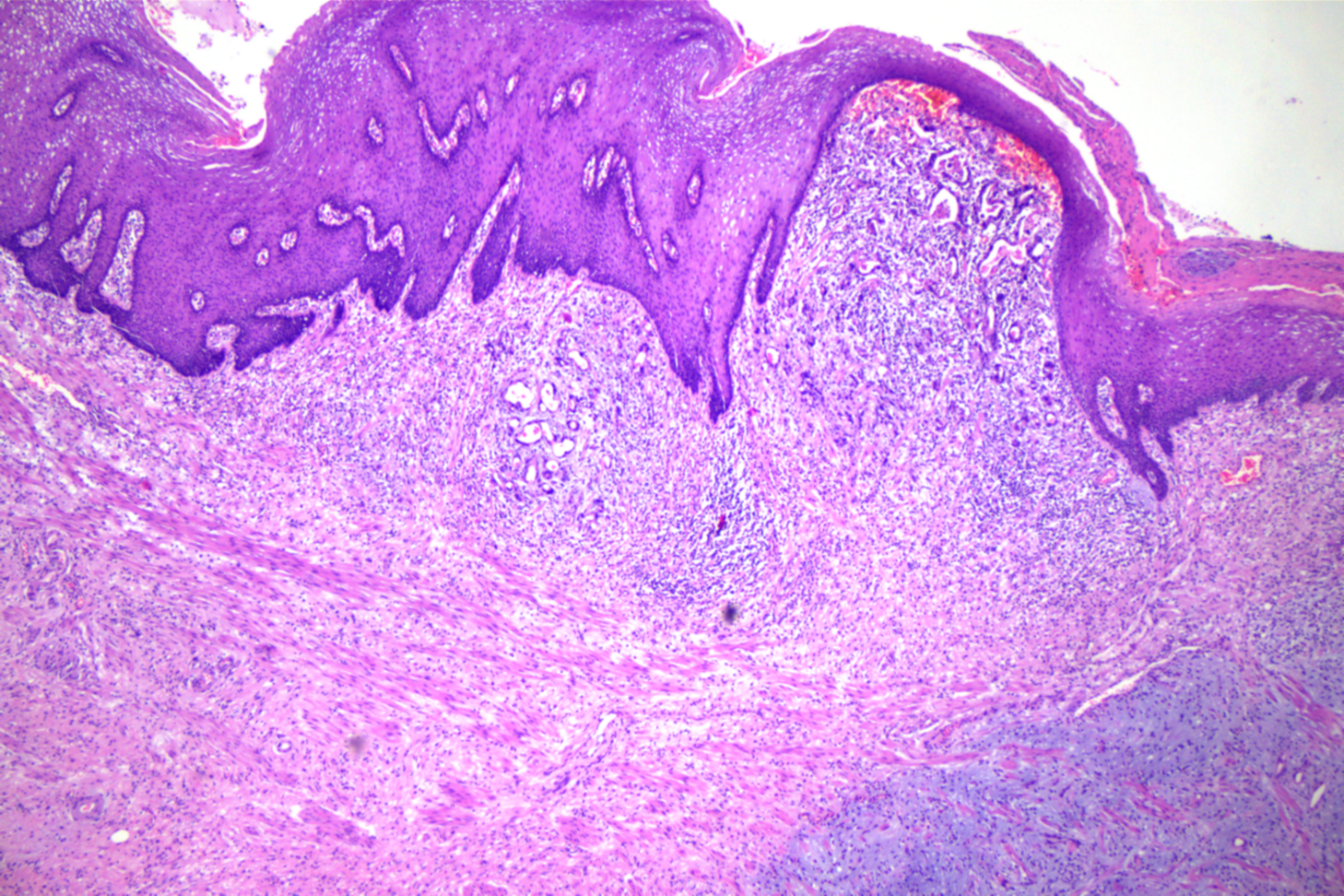  **C** | 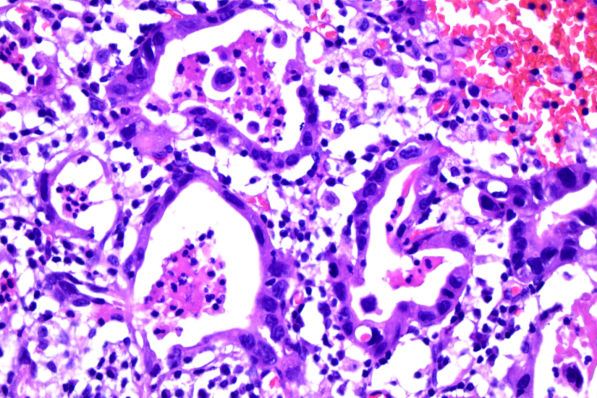  **D** |
| --- | --- |

(C, D) Diffuse/mixed regression pattern. At low magnification (C), residual tumor cells are distributed in an irregular, patchy, and non-uniform manner across the tumor bed, without a clear predominance in either the central or peripheral region. At higher magnification (D), scattered viable tumor glands/cells are seen admixed with fibrosis, inflammatory infiltrates, and treatment-related stromal alterations. This pattern reflects a heterogeneous and dispersed distribution of residual tumor.

| 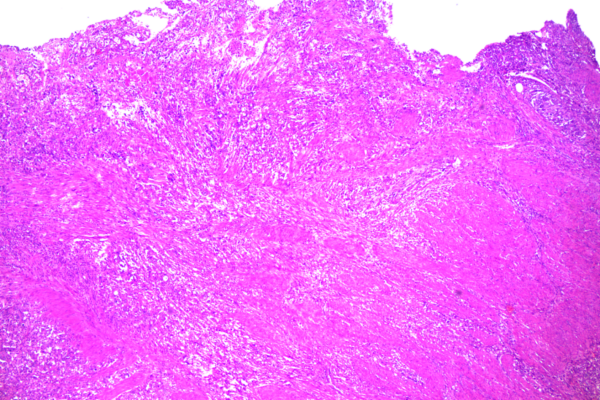  **E** | 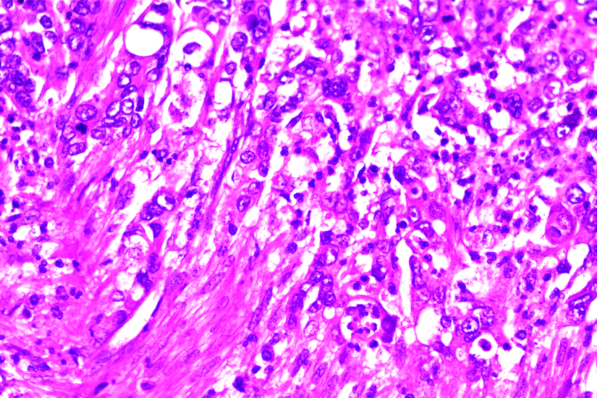  **F** |
| --- | --- |

(E, F) Centrifugal regression pattern. At low magnification (E), the central tumor bed is largely replaced by broad fibrotic/regressive change, whereas residual viable tumor is more evident toward the peripheral aspect of the lesion. At higher magnification (F), residual tumor cells are observed within the peripheral fibrotic stroma, consistent with a pattern in which tumor regression is more pronounced centrally and viable tumor persists mainly at the edge of the tumor bed.
